# Effect of laser power variation on microstructure and properties of selective laser melting coating deposited on TC4 titanium alloy

**DOI:** 10.1371/journal.pone.0356182

**Published:** 2026-09-10

**Authors:** Wei Xu, Gaojian You, Yong Li, Bo Tao, Xiang Li, Guoyang Zhao

**Affiliations:** 1 Chengdu Aeronautic Polytechnic University, Chengdu, China; 2 Jiangxi Key Laboratory of Green General Aviation Power, Nanchang, China; 3 State Key Laboratory of Intelligent Manufacturing Equipment and Technology, Wuhan, China; CHRIST (Deemed to be University), INDIA

## Abstract

The surface properties of aviation titanium alloy directly affect its service safety and reliability, and high-performance coating are essential for its practical application. However, existing studies lack clear guidance on laser power parameters for preparing high-quality TiAl coating on titanium alloy substrates, leaving a research gap in optimizing coating quality and performance. To fill this gap, this work used selective laser melting (SLM) to fabricate TiAl coating on titanium alloy substrates, focusing on the influence of laser power (350–380 W) on coating microstructure, phase composition, elemental distribution and microhardness. SEM, XRD and EDS characterizations were used for systematic analysis. The results show that laser power is the key factor determining coating quality. Severe longitudinal cracks appeared at 350 W and 360 W, leading to poor interfacial bonding and potential coating peeling. The optimal laser power was 370 W, achieving good metallurgical bonding without obvious defects. New Ti_5_Al_11_ and Al_2_Sc phases were formed with a controlled Al/Ti weight ratio of 1:1.8, and the coating reached the highest microhardness of 641.9 HV_0.2_. Excessively high power (380 W) caused numerous cracks and reduced coating performance. This research provides certain technical references and theoretical support for the preparation of TiAl coating on the surface of aerospace titanium alloys.

## 1 Introduction

Titanium alloys boast outstanding specific strength and are extensively deployed in key engineering sectors including aviation, aerospace and weapon systems. They are routinely adopted for the production of aircraft fan blades, compressor disks and diverse hot-section components [1–4]. As modern aviation industry continues to develop, the demanding service conditions place higher requirements on the performance of engine blade materials. However, the native oxide film formed on titanium alloys suffers from low thermal stability at high temperatures. It thus cannot offer reliable shielding for the substrate, which limits the engineering deployment of titanium alloys under high-temperature working conditions. Therefore, surface treatment of titanium alloy materials becomes particularly necessary. Existing mainstream technologies for preparing protective coatings on titanium alloy surfaces include laser cladding, micro-arc oxidation, thermal spraying and thermal diffusion coating [5–9]. Typical coatings can generally be divided into encapsulating MCrAlY (M = Ni, Co, or Ni + Co) coatings and diffusion-type aluminide coatings [10,11]. Yener et al. [12] deposited Ti-Al-based aluminum coatings on alloy surfaces using the welding method, detecting TiAl_3_, TiAl_2_, and TiAl phases on the coating surface. Loskutova et al. [13] prepared diffusion chromium-aluminum coatings, with the coating’s microhardness increasing by 2.3 times compared to the Ti-6Al-4V base alloy. TiAl intermetallic compounds have high strength and hardness, good wear resistance, excellent thermal stability, and oxidation resistance, providing good protection to the base titanium alloy, making them a good choice for titanium alloy surface protective coating [14–17]. Currently, most coatings are air-cooled, which can easily produce titanium oxides. These titanium oxides are intrinsically brittle, which readily induces crack initiation in the coating under slight external loading. This weakens the coating–substrate adhesion and ultimately deteriorates the coating’s protective capability.In the process of preparing coatings using laser cladding technology, a protective gas is generally used to prevent oxidation of the liquid or solid metal at high temperatures, often requiring a gas protection device to be fixed on the laser head [18,19]. Chen et al. [20] prepared composite coatings on a TC4 base using laser cladding, showing that the main physical phases of the four coatings are TiAl, Ti_3_Al, TiC, and Ti_2_AlC, with the hardness of the four coatings being 1.8 to 2.4 times that of the base hardness, with the 15 vol% coatings having the highest hardness. Fatoba et al. [21] improved the mechanical properties and hardness of titanium alloys using laser metal deposition technology, with the formation of Ti_3_Al leading to an increase in the weight percentage of copper, thus enhancing the tensile strength, yield strength, and hardness properties. However, even when the shielding gas system delivers extended protective coverage under high scanning speeds during laser cladding, partial regions of the coating remain exposed to ambient air for cooling. The shielding gas fails to fully block atmospheric oxygen atoms throughout solidification, which prevents complete elimination of brittle oxide inclusions within the coating.

Selective laser melting (SLM) represents the most prevalent metal additive manufacturing technique available today. It features prominent merits including the fabrication of intricate components, high material utilization efficiency and a shortened production cycle.The technology has been relatively mature in the lightweight metal forming field of aluminum alloy, titanium alloy, etc., and has been commercially applied in aerospace, medical, automotive, and other fields [22]. Furthermore, metallic powders can be consolidated within an entirely oxygen-free chamber. A high-purity argon atmosphere of 99.99% effectively suppresses the generation of brittle oxide phases.By using SLM technology to form stable titanium-aluminum intermetallic compounds on the surface of titanium alloy, high melting point and high hardnesscoating can be obtained. The coating can metallurgically bond with the substrate, prolonging the service life of titanium alloy aerospace engine structural components and adapting to a wider range of operating environments. Furthermore, solute atoms undergo solidification under extremely high cooling rates. Long-distance diffusion and atomic rearrangement are suppressed accordingly. This restricts the precipitation of undesirable equilibrium phases and facilitates the formation of metastable phases absent under equilibrium conditions [23]. Carrying out research on the performance of coating formed by laser selective melting of titanium alloys and revealing the formation mechanism of the coating is of great significance for the surface protection and modification of aerospace titanium alloys. The innovative application of laser selective melting (SLM) technology to the in-situ formation of coating on titanium alloys has completed the design and formation preparation of multi-alloyed modified coating on the surface of titanium alloys. Systematically analyzing the influence laws of laser power on the formation behavior of coating on the surface of TC4 alloy. The existing research on SLM has formed a general consensus. Insufficient laser energy input will cause coating bonding defects, while excessive laser energy is prone to induce coating cracking. Unlike investigations into conventional processing parameters, the present work targets a narrow laser power window of 350–380 W. It primarily clarifies how minor power variations regulate the coating’s microstructure and mechanical performance.This has formed a new understanding different from existing research. This work systematically clarifies coating densification behavior, the evolutionary trends of pores and lack-of-fusion defects, as well as the interfacial metallurgical bonding features across this narrow power window.It establishes the phase evolution trends correlated with laser power variation and corresponding microstructural development, and clarifies the inherent correlation between the segregated distribution of Al and the coating microhardness.It upgrades the optimization framework for SLM fabricated coatings, offers new analysis perspectives and technical support. These resources help boost titanium alloy surface performance and guide coating design. Meanwhile, this study provides solid theoretical and experimental references. The references support additive manufacturing of coatings for aerospace titanium alloys.

## 2 Materials, equipment and methods

**Fig 1:**
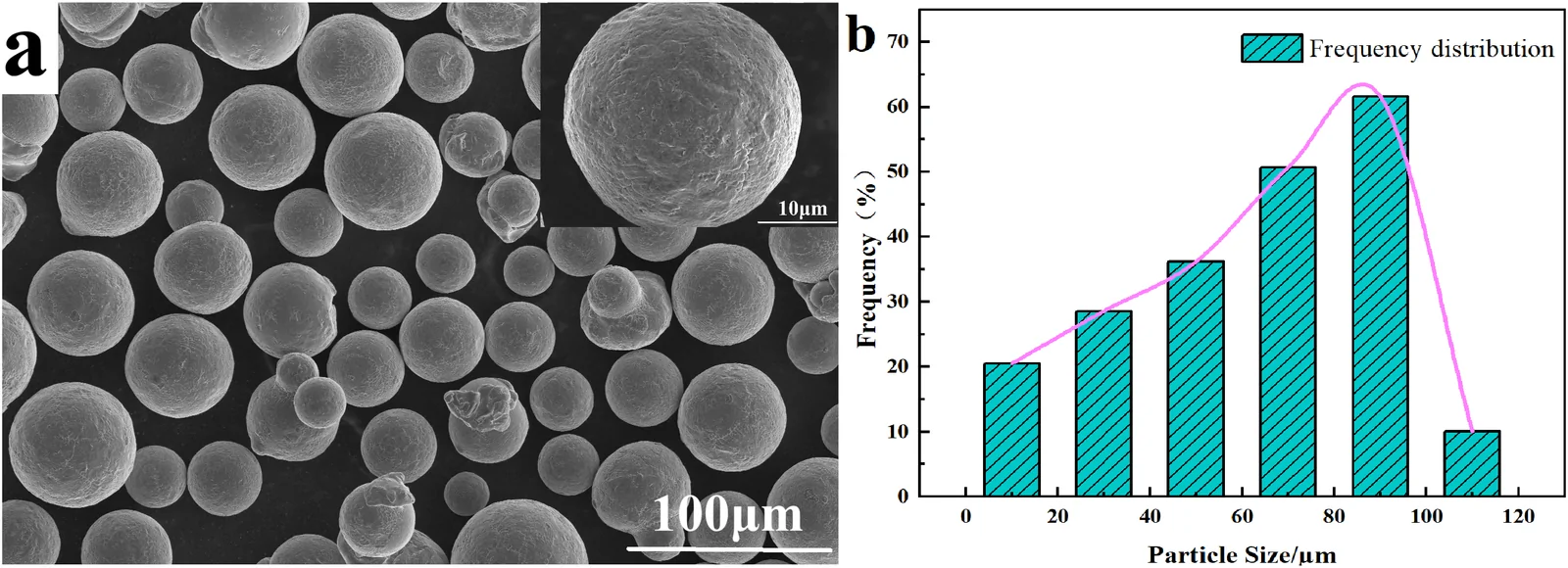
Morphology of AlMgSc powder and distribution of powder particle sizes. (a) Powder morphology; (b) Particle size distribution of the powder.

**Fig 2:**
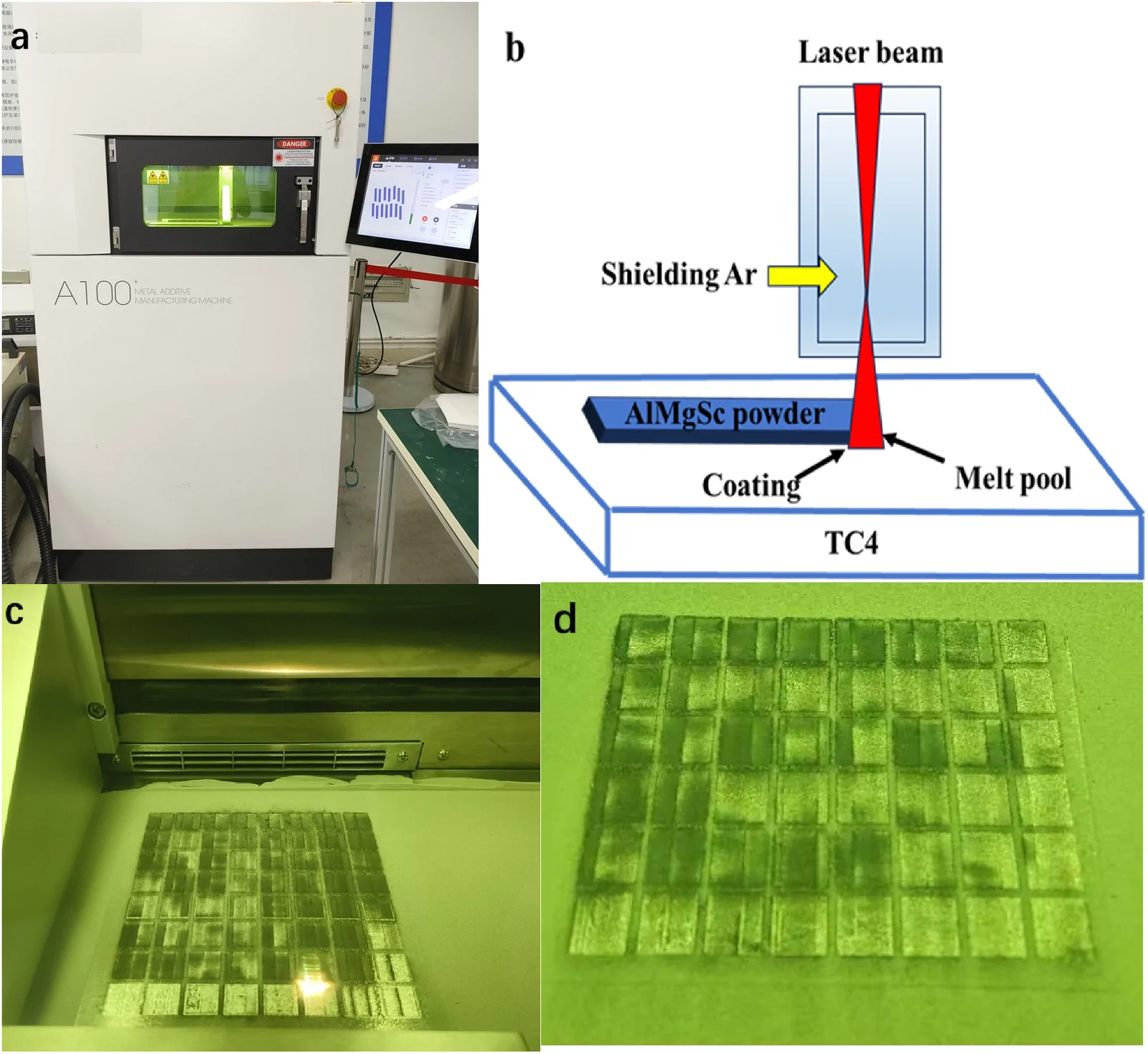
Schematic of experimental equipment, forming principle and printing process. (a) Experimental equipment; (b) Working principle diagram; (c) Coating formation process; (d) Coating solidification process.

Using the DMP Flex350 selective laser melting equipment produced by GF company, equipped with a soft blade powder delivery system, the apparent density is 1.42 g/cm^3^, and the angle of repose is 37°. The surface morphology of the prepared AlMgSc particles was observed using the Thermo Scientific Quattro scanning electron microscope (SEM), as shown in Fig 1. The AlMgSc matrix powder maintained a high degree of sphericity. The powder particle size distribution results measured by the laser particle size analyzer are shown in Fig 1(b), with D10 = 20.5 µm, D50 = 36.2 µm, and D90 = 61.1 µm, can achieve uniform spreading and recycling of powder, providing reliable hardware support for high-precision control of the forming process. The core component of the equipment adopts a 500W fiber laser, with a spot diameter of 100μm, capable of outputting stable laser energy. With an adjustable powder spreading thickness of 10–100μm and a scanning speed of up to 7m/s, the equipment can accurately control the dynamic behavior of the melt pool. The built-in atmosphere control system of the equipment can ensure that the oxygen volume fraction in the processing environment is maintained at ≤0.0025%, effectively avoiding the oxidation of metal powder in a high-temperature environment and ensuring the purity of the formed part. The experimental equipment, forming principle, and printing process are shown in Fig 2.

The test material selected is the AlMgSc powder prepared by the vacuum gas atomization method, which has excellent sphericity and smooth surface morphology, meeting the stringent requirements for powder in the SLM process; the chemical composition of the powder is detailed in Table 1, with precise proportions of various alloying elements, providing an important basis for the control of the subsequent formed part properties.

**Table 1:** Chemical compositions of AlMgSc (mass fraction, %).

Main component /%			Impurity /%, Not more than			
Al	Mg	Sc	Zr	Si	Cu	Fe
Allowance	5.09	0.84	0.34	0.03	0.01	0.07

In the experiment, the TC4 base parts to be processed were fixed on the worktable of the forming equipment. This fixation method is closely related to the printing process and the tools and equipment, and directly affects the forming accuracy of the coating and the quality of the interface bonding. The fixation of the substrate is the prerequisite for layer-by-layer melting and accumulation, which can prevent the coating from shifting and ensure uniform thickness. Using the above-mentioned AlMgSc metal powder, a coating was formed by printing in a layered melting and stacking manner, with a geometric dimension set at 15 mm × 15 mm × 1 mm. This parameter is determined through reasoning based on the test purpose, equipment performance, and subsequent detection requirements. A too small size will affect the subsequent performance detection, while a too large size will increase the consumption of materials and time and is prone to causing defects. This size is suitable for the printing accuracy of the equipment, can precisely achieve the coating thickness, ensure an appropriate contact area of the substrate, and provide suitable samples for subsequent performance detection. The main forming process parameters are detailed in Table 2. The samples were cut using an electrical discharge wire cutting machine, and the cross-section of the base and coating was polished on sandpaper, followed by immersion corrosion in a solution with a volume ratio of HF, HNO_3_, and H_2_O of 1:2:7 to prepare metallographic samples. Meanwhile, the microscopic morphology of the coating and base interface was analyzed using a JSM-IT500 scanning electron microscope (SEM) equipped with an energy dispersive spectrometer (EDS), and the distribution characteristics of elements such as Ti, Al, Mg, and Sc in the interface transition zone were explored by line scanning analysis. The phase composition of the coating and interface regions was determined using a Rigaku Ultima IV X-ray diffractometer (XRD). Microhardness testing was performed using an HV-1000A Vickers hardness tester, with continuous indentation points every 50μm in the coating and base interface region, a load of 1.96N (corresponding to 0.2kgf), a holding time of 10s, and 10 valid data points per test group. In this experiment, three parallel samples were independently prepared for each laser power condition, ensuring the reliability and repeatability of the test data. By precisely controlling and characterizing the process parameters, the strengthening mechanism of the coating formed by SLM on the TC4 surface with a laser power gradient control is deeply revealed, providing a rigorous experimental basis.

**Table 2:** SLM main test parameters.

Sample No.	Laser power /W	Scanning speed/mm/s	Spot diameter /mm	Strip overlap /mm	Layer thicknes/mm
1	350	1000	0.1	−0.02	0.03
2	360	1000	0.1	−0.02	0.03
3	370	1000	0.1	−0.02	0.03
4	380	1000	0.1	−0.02	0.03

## 3 Results and discussion

### 3.1 Macroscopic and microscopic

**Fig 3:**
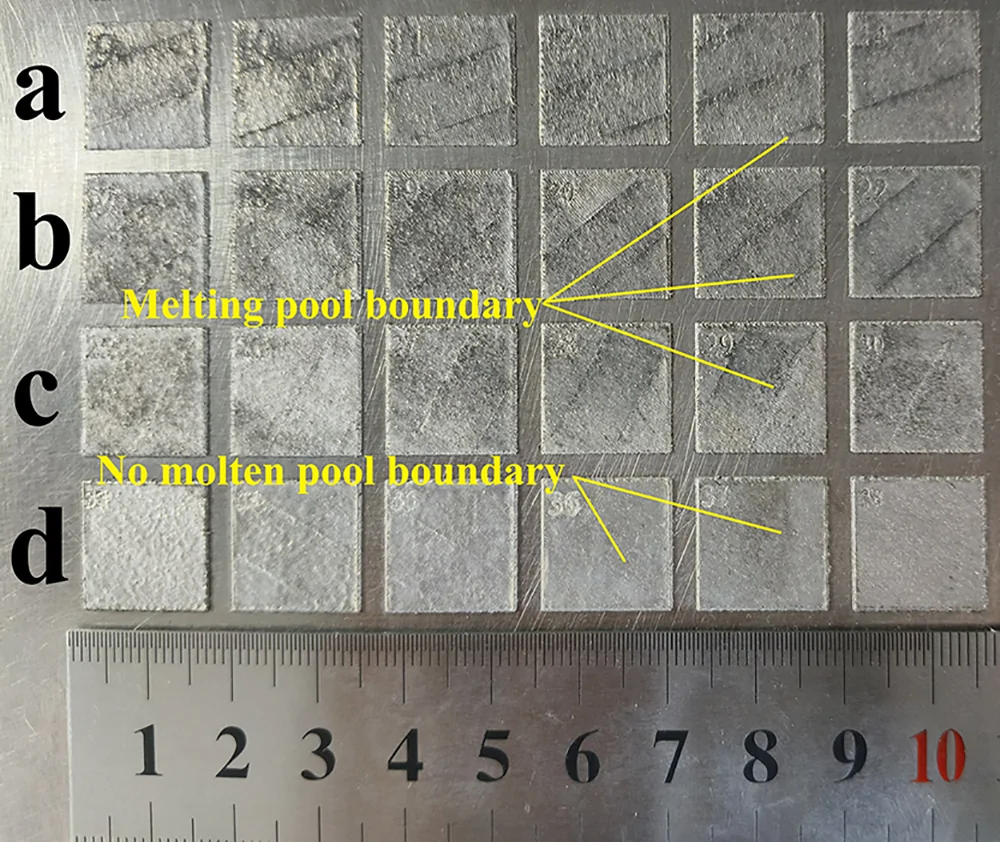
Macroscopic morphology of coating formed with different laser powers (a) 350W; (b) 360W; (c) 370W; (d) 380W.

Fig 3 shows the macroscopic morphology of SLM formed coatings on TC4 surface under different laser power conditions, where (a) is 350W, (b) is 360W, (c) is 370W, and (d) is 380W. The outer surface of all coatings presents a uniform bright white appearance consistent with aluminum alloy, indicative of superior surface smoothness and gloss. The surface finish reaches a high level, indicating that within this power range, the interaction between laser energy and material can achieve stable melting and solidification processes, forming dense and continuous coating structures. The interface reaction zone is the selected titanium-aluminum intermetallic compound protective coating. Macroscopic observations reveal negligible morphological variations among coatings produced at different laser powers. The applied laser energy density falls within a proper window. This window enables full melting of TC4 powder and sustains moderate melt pool fluidity. The melt pool can quickly spread and solidify uniformly, maintaining consistency in the macroscopic features of the coating surface. The laser melt pool boundary on the outer surface of the coating under 380W power is relatively blurry compared to coating under other power conditions, appearing smoother. Higher laser power lowers the surface tension of liquid metal and improves fluidity. The molten pool thus spreads and fully fuses for a longer duration prior to solidification. Overlap marks between neighboring molten pools become less prominent, and a smoother surface morphology is consequently formed. This accounts for the improved surface quality. The rapid solidification process also reduces the generation of surface defects.

**Fig 4:**
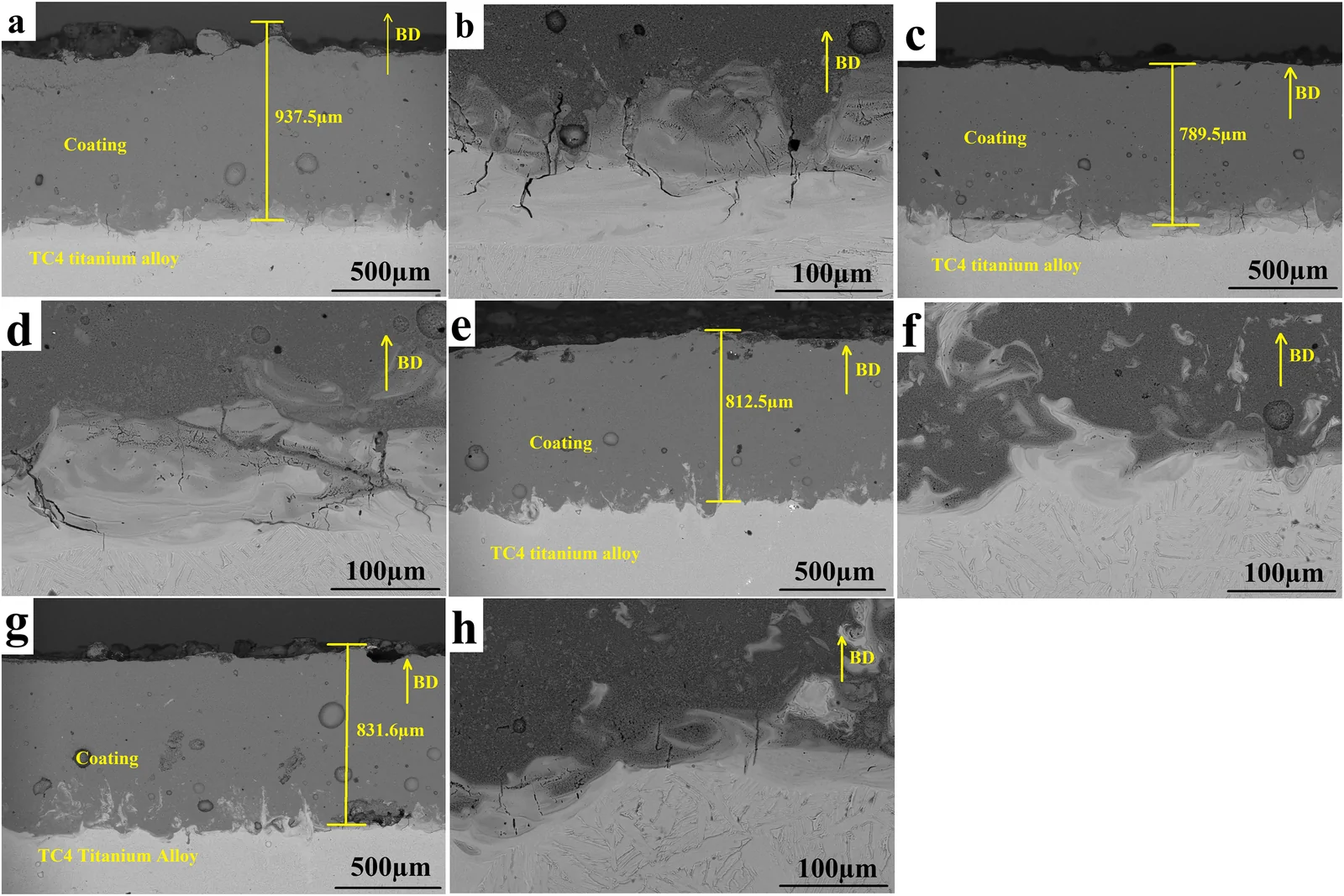
Coating formed by different laser powers. (a)350W, 200 × ; (b)350W, 1000 × ; (c)360 W, 200 × ; (d)360 W, 1000 × ; (e)370 W, 200 × ; (f)370 W, 1000 × ; (g)380 W, 200 × ; (h)380 W, 1000 × .

Figs 4(a) and 4(b) show the formed coating when the laser power is 350W, building direction (BD) is the construction direction. The thickness of the coating can reach approximately 937.5 µm. For each laser power group, three independent parallel samples were prepared. Each sample was cut to prepare cross-sectional metallographic specimens. For each sample section, three observation areas were selected at equal intervals, and 5 coating thickness readings were randomly collected from each area. The average coating thickness value of each group was calculated as the final coating thickness value. The outer layer of the coating is the deposited AlMgSc layer, and the interface between the coating and the substrate is the Ti-Al reaction zone and the intermetallic compound transformation zone. There are many longitudinal cracks perpendicular to the interface in the area where the coating is bonded to the substrate, and they extend to the base alloy. The surface flatness of the coating is poor. The direct influence of the laser energy density on the behavior of the molten pool, and the laser power and scanning speed as the key regulating parameters of the energy density, are calculated according to formula (1), directly determining the melting and solidification behavior of the molten pool, and thereby affecting the forming quality of the coating.

**Fig 5:**
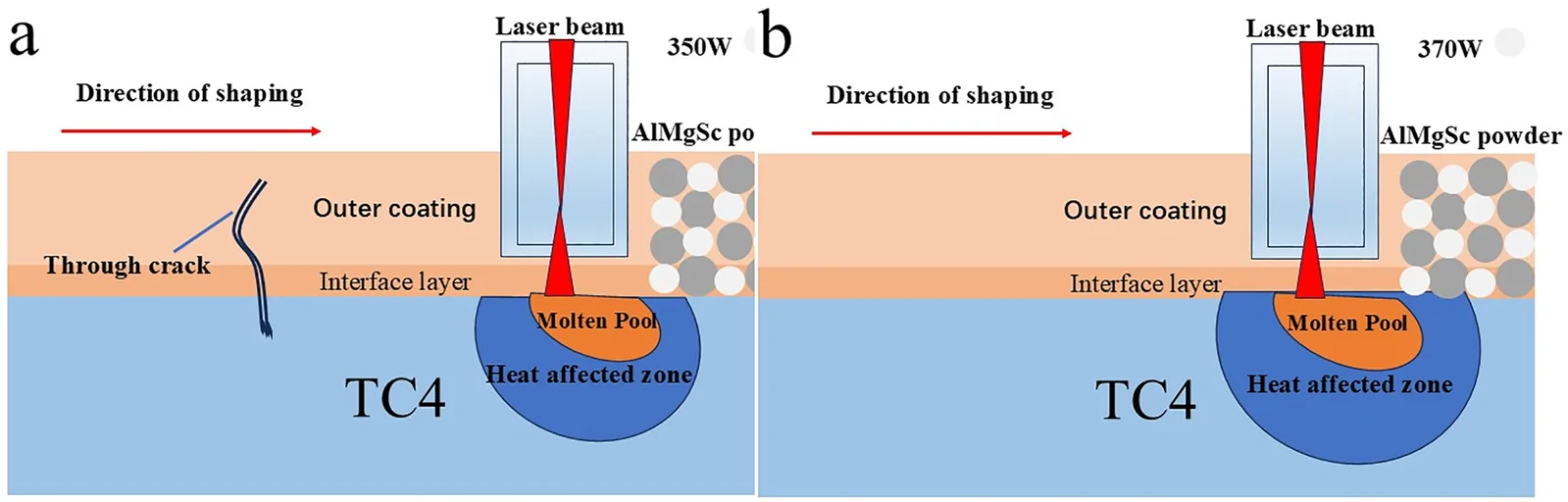
Schematic diagram of the coating forming principle at different laser powers. a. 350W, b. 370W.

E: represents the laser energy density (J/mm^3^), P: denotes the laser power (W), t: indicates the thickness of the powder laying layer (mm), s: stands for the scanning spacing (mm), and v: signifies the scanning speed (mm/s).When the laser power is 350W, the effective energy input to the molten pool is insufficient, leading to the energy density of the molten pool being at a low level. The principle of coating formation is illustrated in Fig 4a, the possible reasons might be: the heat-affected zone is also relatively small.Thermal convection inside the melt remains weak. It slows the diffusion speed of solute atoms. The melt cannot fully spread across the substrate surface, and convective agitation fails to eliminate localized compositional segregation; low laser power accelerates the solidification rate of the molten pool, shortening the time for melt flow and composition homogenization, further exacerbating the degree of composition segregation. During solidification, mismatched thermal expansion coefficients exist between the coating and substrate. Low laser power generates an uneven temperature gradient across the molten pool, alongside extremely fast cooling rates. This ultimately manifests as longitudinal cracks perpendicular to the interface, with insufficient spreading leading to a visibly uneven coating surface [24]. Fig 4(c) and 4(d) show the coating at a laser power of 360W, with a thickness reduced to about 789.5µm, coating thickness fluctuates owing to variable sputtering power and inconsistent melt penetration depth. Besides longitudinal cracks, these factors also induce uneven coating thickness, other directionally random cracks appear, extending into the substrate in several places. The increase in energy density alters the morphology of the molten pool; on one hand, changes in solidification rate cause uneven distribution of thermal stress, and on the other hand, fluctuations in energy input decrease the stability of the molten pool spread, but the surface flatness of the coating improves compared to that at 350W. Fig 4(e) and 4(f) depict the coating formed at a laser power of 370W, with a thickness of about 812.5µm, and no obvious cracks were found, indicating a significant improvement in coating quality. The energy density is in a more optimal range, allowing for sufficient melting and uniform spreading of the molten pool, with a reasonable distribution of the temperature gradient during the solidification process. The Fig shows a good metallurgical bonding between the coating and the substrate, with the principle of coating formation illustrated in Fig 5(b). The heat-affected zone is larger compared to that at 350W, thus preventing crack propagation into the substrate. Fig 4(g) and 4(h) show the coating at a laser power of 380W, with a thickness of about 831.6µm. The excessively high power causes the energy density to exceed the optimal range. Excessive energy concentration triggers local overheating at the melt pool center. This generates concentrated thermal stress at the interface as solidification proceeds. Although the surface flatness of the coating is decent, the presence of cracks severely affects the coating quality.

The coating thickness does not have a simple monotonic relationship with the laser power. As the laser power gradually increases, the coating thickness first decreases and then increases. This phenomenon can confirm that there are complex dynamic behaviors within the molten pool. When the laser power is low, the convective movement of the molten pool is driven by the surface tension gradient, and the melt is transported outward, allowing the coating to fully spread and accumulate. When the laser power is increased to 360 W, the laser energy density significantly increases, and the local temperature of the molten pool approaches or even reaches the critical temperature for titanium alloy evaporation. At this time, metal vapor will violently erupt, and a recoil pressure directed towards the molten pool interior will be generated. This pressure will squeeze the free surface of the molten pool, and at the same time, a large number of tiny droplets will splash. Some liquid metal will separate from the molten pool in the form of vapor or droplets, ultimately causing a significant decrease in the effective thickness of the solidified coating. When the laser power is further increased to 370 W and 380 W, the molten pool transitions to a different operating mode. The effective absorption rate of laser energy is significantly improved, and the melting volume of the melt rapidly increases. Under such conditions, the amplified recoil pressure intensifies the internal stirring motion inside the molten pool, drives the molten material from the pool bottom toward the surface, and further facilitates lateral spreading of the coating. Metal vaporization loss still occurs within this laser power window, yet the melt replenishment rate exceeds the material loss rate, which halts the decline of coating thickness and enables its gradual recovery. The coating thickness under the 370 W and 380 W power conditions has not yet returned to the thickness level corresponding to the 350 W power. This indicates that in the high laser power range, the metal evaporation effect has not disappeared, and the convective effect of the molten pool has formed a new dynamic balance. The non-monotonic variation of coating thickness primarily arises from the shift in laser interaction mechanism from heat conduction mode to keyhole mode, which triggers this nonlinear dynamic response.

### 3.2 Phase analysis

**Fig 6:**
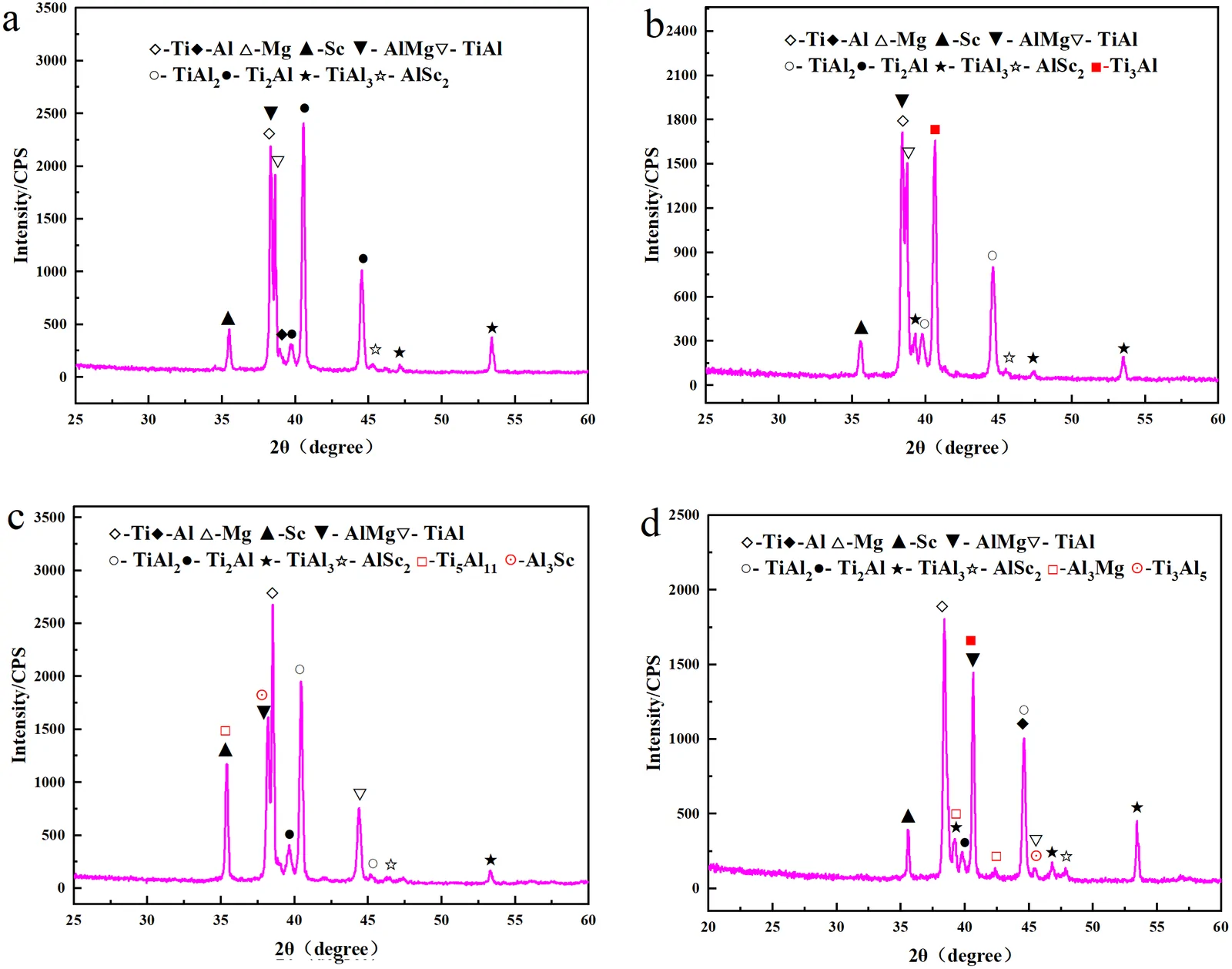
Physical phase analysis of the coating bond at different laser powers. (a) 350W; (b) 360W; (c) 370W; (d) 380W.

**Fig 7:**
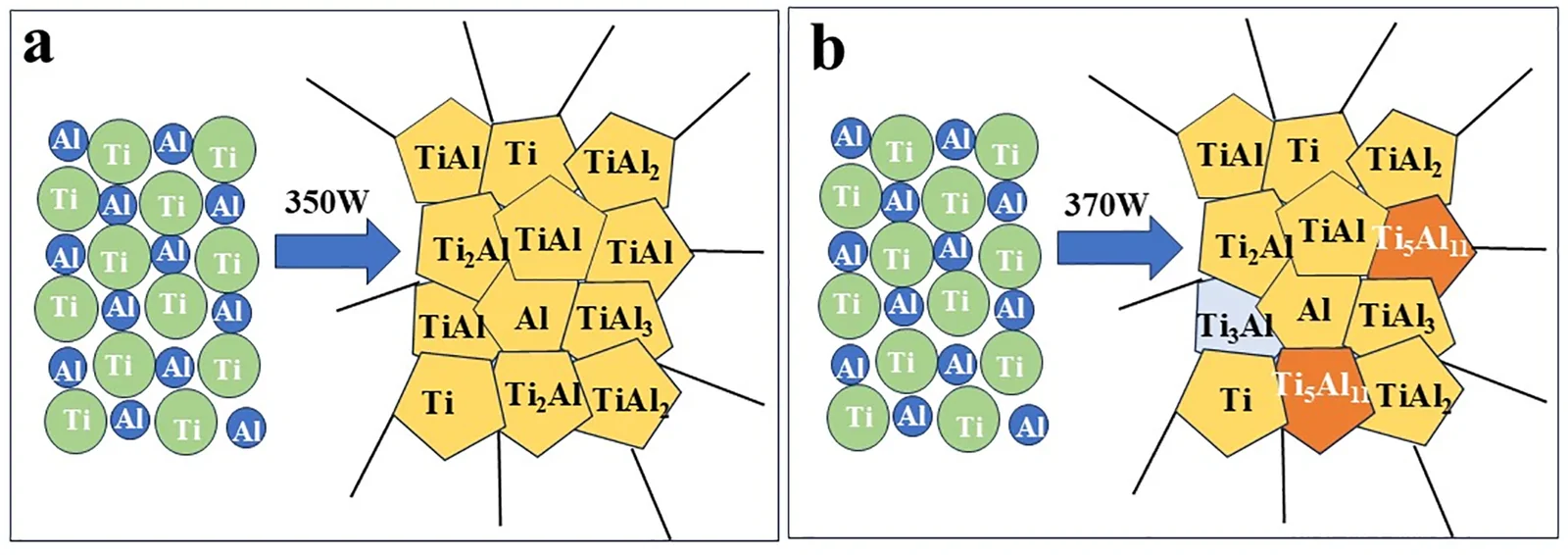
Principle of coating formation with different power shaping. (a) 350W; (b) 370W.

Fig 6 shows the phase detection results of the bonding zone of the coating under different laser powers. At a power of 350W, the coating detected TiAl, TiAl_2_, Ti_2_Al, TiAl_3_, AlMg, as well as elemental Ti, Al, Mg, and Sc. At this time, the molten pool temperature was not high, and the degree of atomic migration and diffusion was weak. The formation of new phases must meet thermodynamic conditions, with the Gibbs free energy change ΔG being less than zero, corresponding to the formula ΔG = ΔH – TΔS. Low power brought a low-temperature environment, and the atomic kinetic energy was insufficient. The negative value of the system ΔG was small, not enough to significantly generate intermetallic compounds, so the types of new phases generated at this power were relatively few. When the power was increased to 360W, the laser energy density significantly increased, and the driving force for atomic diffusion became stronger. The overall temperature of the molten pool rose, widening the temperature range that could satisfy ΔG < 0, and the Ti and Al atoms obtained sufficient activation energy. In addition to the phases detected at 350W, two new phases, Ti_3_Al and AlSc_2_, were detected. According to thermodynamic calculations, the molten pool temperature reached the nucleation requirements for Ti_3_Al, and the ratio of Ti and Al atoms in the system was close to 1:3. The formation mechanism of AlSc_2_ was consistent, with the diffusion efficiency of Sc and Al improving, allowing the system ΔG to drop to a negative value and form the phase successfully. When the power was increased to 370W, the laser energy supply was further adapted, the temperature distribution of the molten pool became more uniform, and the atomic diffusion coefficient increased. In addition to the phases detected at 360W, two new phases, Ti_5_Al_11_ and Al_2_Sc, were added. At this time, the molten pool temperature met the thermodynamic standard for the nucleation of Ti_5_Al_11_, with ΔG less than zero and sufficient phase formation driving force; higher power extended the diffusion path of Sc and Al atoms, increasing the probability of atomic collisions, and facilitating the stable growth of Al_2_Sc [25–27]. When the power reached 380W, excessive input of energy caused local overheating of the molten pool. Although the atomic diffusion rate further increased, the high temperature changed the original phase equilibrium state of intermetallic compounds. The values of enthalpy change ΔH and entropy change ΔS fluctuated and shifted, and the nucleation temperature range of ΔG < 0 for each phase changed accordingly. In addition to the phases detected at 360W, two new phases, Ti_3_Al_5_ and Al_3_Mg, were added. Thermodynamic analysis determined that the local high temperature matched the nucleation temperature of Ti_3_Al_5_; the high-temperature environment also caused local excessive accumulation of Al atoms. Magnesium atoms moved rapidly under high temperature, disrupting the original atomic distribution ratio, and could combine with Al in a fixed proportion, with the system ΔG less than zero, completing the nucleation and growth of Al_3_Mg [28,29].The formation principles of coating at different power levels are illustrated in Fig 7, where the differences in elemental migration and distribution balance at various power levels can be observed. As the power increases, the bonding between titanium and aluminum atoms becomes tighter, as shown in Fig 7(b), providing a foundation for the formation of new phases.

**Fig 8:**
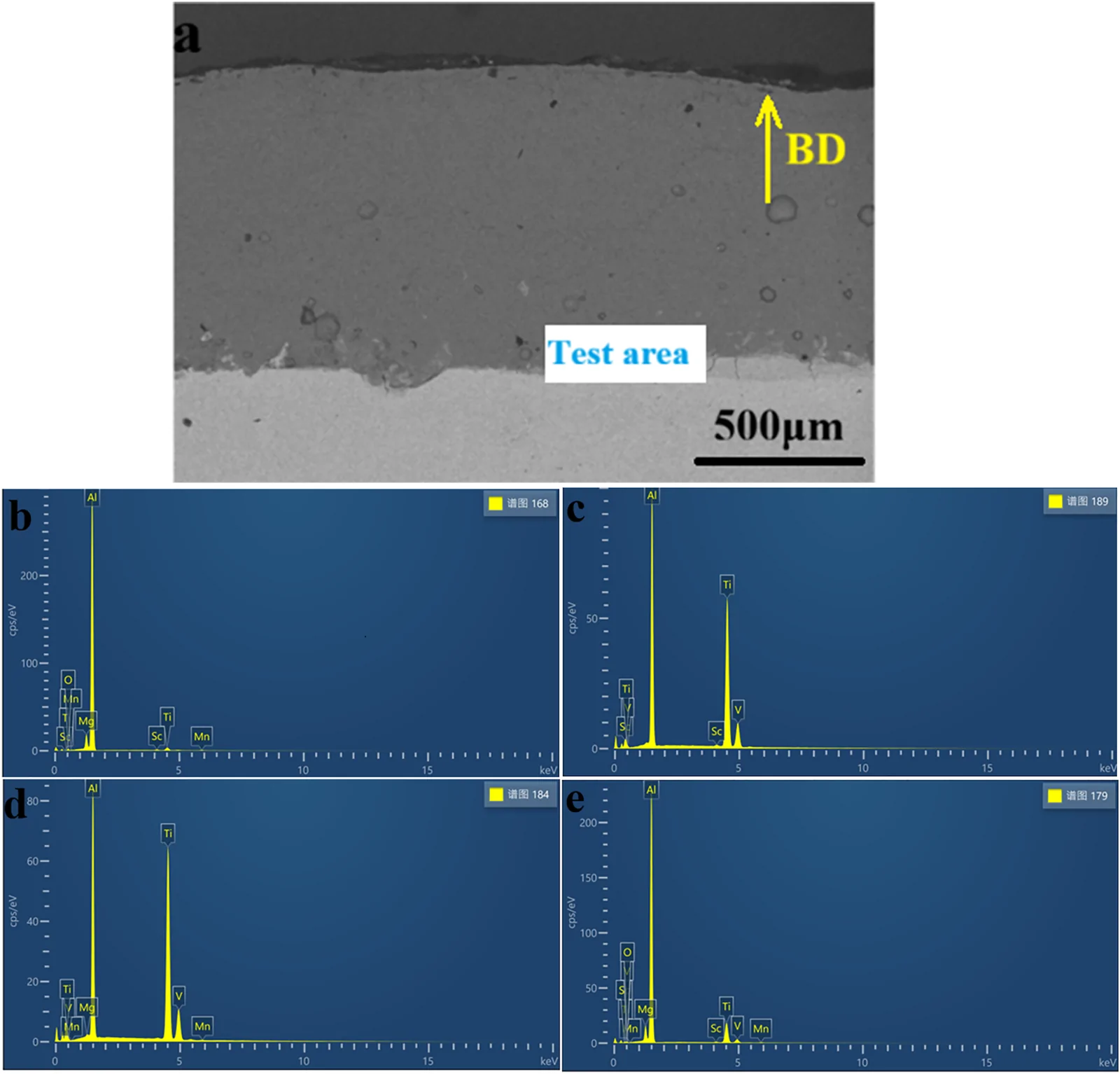
Coating EDS shaped by different laser powers. (a) EDS test area; (b) EDS spectral analysis at 350W; (c) 360W; (d) 370W; (e) 380W.

As shown in Fig 8 and Table 3, EDS spectrum analysis of the coating bonding area under different laser power levels is presented, and the elemental quantification of the coating bonding area is conducted. When the laser power is 350W (as shown in Fig 8b and Table 3), the input laser energy is relatively low, and the overall temperature of the molten pool is at a relatively low level. The low temperature cannot provide sufficient energy for the interfacial diffusion of titanium atoms in the base material, and the diffusion driving force of titanium atoms is low. At the same time, the fluidity of the molten pool in the low-temperature state is weak, and the migration movement of coating Al atoms is significantly restricted, making it difficult for them to penetrate the interface and diffuse towards the titanium alloy substrate. Under the effect of dual diffusion limitations, a phenomenon of a large accumulation of Al elements occurs in the bonding zone. In this condition, the mass percentage of Al and Ti elements in the bonding zone is 24:1 [30,31]. After the laser power is increased to 360W(as shown in Fig 8c and Table 3), the laser energy input is improved, and the temperature of the molten pool rises synchronously. The molten pool after heating provides sufficient diffusion kinetic energy for the Ti atoms to stably diffuse from the base material to the coating area; at the same time, the fluidity of the molten pool improves significantly with the increase in temperature, and the Al atoms in the coating can smoothly migrate towards the base material side. The efficiency of bidirectional atomic diffusion is simultaneously enhanced, effectively strengthening the interdiffusion effect of Ti and Al atoms, and the imbalance of the distribution of interface elements is significantly improved. The Al/Ti mass percentage drops to 1:1.5, indicating that within this medium-low power range, appropriately increasing the laser power can effectively optimize the interface mass transfer behavior and promote the gradual balance of the element distribution at the coating and base material interface [32]. When the laser power is further increased to 370W(as shown in Fig 8d and Table 3), the laser energy input reaches the optimal state, and the molten pool can maintain a stable high-temperature environment for a long time. The stable high-temperature condition combined with the directional convection movement of the molten pool improves the distribution state of Al atoms, making the Al elements more evenly distributed in the bonding zone. At this time, the element concentration gradient forms a dynamic equilibrium state, and the Al/Ti mass percentage is regulated to 1:1.8, which is the preferred power condition for the combination of TiAl coating interface elements [33]. At a laser power of 380W (as shown in Fig 8e and Table 3), although the diffusion ability of Ti atoms remains strong, strong convection of molten material at high temperatures leads to secondary Al atom enrichment near the interface, resulting in the Al/Ti mass percentage rising again to 4:1, revealing the reverse impact of excessive power on the equilibrium of elemental distribution.

**Table 3:** EDS spectrum comparison.

Element Wt%	Ti	Al	Mg	Sc
1-350W	3.46	88.42	4.09	0.65
2-360W	57.88	39.08	0	0.38
3-370W	62.69	34.00	0.27	0
4-380W	18.53	73.86	3.97	0

### 3.3 Microhardness

**Fig 9:**
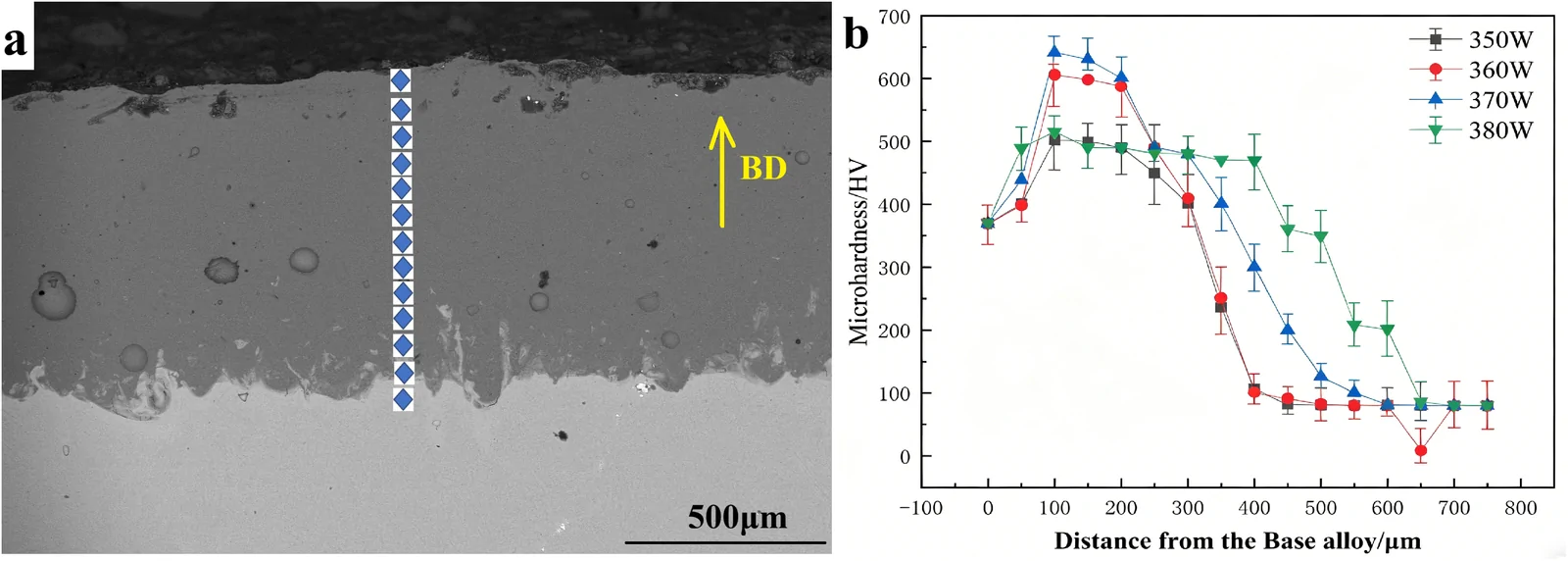
Cross-sectional hardness analysis of coatings formed with different laser powers. (a) Coating hardness distribution area; (b) Coating hardness area analysis.

Fig 9 shows the microhardness analysis results of the coating bonding area under different laser power. During the SLM forming process,Ti and Al elements primarily interact at the bonding interface of coating and substrate, where intermetallic compounds are also mostly formed. Accordingly, the coating reaches its maximum hardness near the bonding interface, with hardness values gradually declining toward the coating outer layer; the outermost coating region consists of unreacted raw aluminum alloy free from thermal reactions, so its hardness shows no obvious discrepancy relative to the bulk aluminum alloy substrate. At 350W, the maximum hardness of the coating is only 502.4HV_0.2_, which is only a 35.8% increase compared to the 370HV_0.2_ of the substrate TC4 alloy, indicating limited improvement in hardness. The lower laser power results in insufficient energy input, low driving force for the diffusion of Ti-Al atoms, and inadequate atomic diffusivity, leading to the formation of only a small amount of low-bond energy intermetallic compounds at the bonding site, with coarse grain sizes that restrict the hardness increase;the higher the binding energy of the intermetallic compounds and the more uniform the precipitation, the more significant the increase in coating hardness [34,35]. At 360W, the increase in laser power results in greater energy input, significantly enhancing the diffusion capacity of Ti-Al atoms, extending atomic migration distances, and increasing collision probabilities. This promotes increased precipitation of intermetallic compounds at the bonding interface and optimizes their phase constitution, which yields mild grain refinement and further elevates the interfacial bonding energy. The maximum hardness of the coating increases to 598.6HV_0.2_, with the peak hardness still concentrated near the bonding site, in accordance with the hardness distribution pattern. At 370W, the laser power reaches a more optimal range for Ti-Al atomic diffusion, with sufficient energy input that does not cause local overheating, allowing for full and uniform atomic diffusion. A large amount of intermetallic compounds is generated at the bonding site, while grain size is effectively refined, resulting in a significant increase in hardness, with the maximum hardness of the coating reaching 641.9HV_0.2_, which is the peak value among all power levels. At 380W, reducing the maximum hardness of the coating to 515.5HV_0.2_; however, the high energy input breaks through the spatial limitations of atomic diffusion, promoting the long-range diffusion of Ti-Al elements and widening the distribution range of intermetallic compounds in the bonding area until it approaches the outer side of the coating, at which point the hardness gradually decreases to a level comparable to that of the aluminum alloy [36–38].

Xu et al. [39] employed pressure-assisted high-temperature thermal diffusion to fabricate coating on titanium alloy substrates. Through the synergistic regulation of external pressure, this method greatly boosts the bonding strength at the coating-substrate interface, alongside simultaneous improvements in microstructural uniformity and comprehensive mechanical properties.Under optimized conditions (750 °C, 160 Pa), the resulting coating exhibited a relatively thin total thickness and a surface microhardness of 378.4 HV. However, a distinct interface was observed between the outer layer and the transitional bonding layer, accompanied by pronounced delamination—indicating compromised structural integrity and reduced service reliability. Yue et al. [40] applied magnetron sputtering to deposit protective coating, achieving a maximum microhardness of 484 HV,a notable improvement in hardness. Nevertheless, this technique suffers from inherent practical constraints, including high capital and operational costs, low material utilization efficiency, and substantial target erosion losses. Li et al. [41] integrated electromagnetic field assistance into the coating fabrication process, reporting elevated interfacial hardness—particularly under optimized current parameters—where the peak coating hardness reached 433.4 HV. Yet, the resultant coating exhibited excessive thickness, introducing unnecessary mass loading on the substrate and potentially accelerating interfacial stress accumulation during thermal or mechanical cycling, thereby undermining long-term protective efficacy. The performance comparison of the coating formed by SLM with other coating processes is shown in Table 4. In contrast, this study utilizes SLM to fabricate composite coating. SLM offers three key advantages: (i) high material utilization (>90%), minimizing raw material waste and enabling more predictable and scalable cost control; (ii) exceptional spatial and dimensional controllability—allowing precise tailoring of coating thickness and geometry to match functional requirements, thus avoiding performance degradation caused by either under-coating (insufficient protection) or over-coating (excessive residual stress or spallation risk); and (iii) superior microstructural uniformity, with continuous, dense, and defect-free coating- substrate interfaces – free from observable delamination, cracking, or porosity. Consequently, coatings fabricated via SLM strike an optimal balance between structural integrity and service adaptability, exhibiting remarkable technical advantages and great scalable potential for advanced surface engineering of titanium alloys.The SLM forming TiAl coating still has certain technical limitations. It is only suitable for the preparation of coating on planar, regular surfaces and is difficult to adapt to complex geometric components such as curved surfaces and irregular structures. When dealing with parts with variable contours and complex configurations, the layer-by-layer powder deposition process of SLM will encounter problems such as uneven powder spreading, poor adhesion of the fusion zone, and insufficient continuity of coating formation. This makes it impossible to ensure the uniform preparation and stable bonding of coating surfaces on complex components, which to some extent limits the engineering applicability of this process. In the future, optimization exploration can be carried out in combination with the laser powder feeding cladding process. By leveraging the flexible processing characteristics of synchronous powder feeding, it can flexibly adapt to various irregular curved surfaces, internal cavities, and complex-shaped parts for surface coating processing. This technique delivers superior processing flexibility, as its melt scanning path can be adjusted in real time to match component geometries and support the integrated, continuous fabrication of TiAl coatings over complex-shaped part surfaces.This effectively compensates for the application shortcomings of the SLM process in processing complex-shaped workpieces and further expands the practical application scenarios of titanium alloy irregular component surface modification technology.

**Table 4:** Comparison of the performance of TiAl coating formed by SLM with those obtained by other processes.

Forming process	Microstructure	Hardness	Thickness
SLM-formed Ti-Al coating	No obvious cracks	641.9	Controllable
Pressure-assisted Ti-Al coating	Too much pressure, causing the coating to peel off.	378.4	Thicker
Magnetron sputtering coating	No obvious defects	484	–
Electromagnetic field-assisted Ti-Al coating	The defect has been suppressed	433.4	Thicker

## 4 Conclusion

This paper focuses on the multi-alloy design concept of titanium alloy surface modification coating, conducting targeted research around the TC4 matrix. It systematically analyzes the microstructure characteristics and comprehensive performance changes of coating prepared by selective laser melting technology under different laser power conditions. This research is based on the collaborative design concept of multi-component alloys, accurately revealing the mechanism of differential evolution of microstructure caused by laser power fluctuations, clarifying the intrinsic relationship between process parameters, microstructure and service performance, and forming a controllable preparation method for SLM coating suitable for the titanium alloy matrix. Provide new theoretical support and practical references for the parameter optimization and performance regulation of titanium alloy surface strengthening processes.

(1) When the laser power is 350W and 360W, longitudinal cracks can be observed in the bonding area between the coating and the substrate, extending to the substrate alloy. At 370W laser power, the optimal thickness is about 812.5 µm, the coating is well bonded to the substrate with no obvious cracks, and the maximum hardness at the interface reaches 641.9HV_0.2_, significantly improving the quality of the coating. When the laser power is 380W, excessive power exceeds the optimal range, although the surface roughness of the coating is acceptable, the presence of numerous longitudinal cracks severely affects the coating quality.
(2) Under different laser powers, XRD tests on the TC4 substrate surface combined with the SLM coating show: at 350W, the main phases detected in the coating are TiAl, TiAl_2_, Ti_2_Al, TiAl_3_, AlMg, as well as elemental Ti, Al, Mg, and Sc; at 360W, a new phase Ti_3_Al and AlSc_2_ are detected; at 370W, in addition to the phases detected at 360W, new phases Ti_5_Al_11_ and Al_2_Sc are observed.
(3) EDS spectra testing of the coating bond area at different laser powers shows: at 350W, the weight percent of Al/Ti in the coating reaches 24:1; at 360W, the extent of Ti/Al mutual diffusion significantly increases, and the Al/Ti weight percent decreases to 1:1.5; at 370W, the energy input is further optimized, adjusting the Al/Ti weight percent to 1:1.8, reflecting the synergistic and stable interdiffusion of elements at this power level; at 380W, excessive energy leads to local overheating of the melt pool, causing the Al/Ti weight percent to rise again to 4:1.

Meanwhile, this technology is more suitable for the coating processing of planar and regular components. Subsequently, based on the laser synchronous powder feeding cladding process, further exploration can be carried out to break through the limitations of the geometric shape of the workpiece and achieve high-quality preparation of coating on the surfaces of complex-shaped components, further expanding the application scope of titanium alloy surface modification technology.
