# Supplementary material for: Effect of laser power variation on microstructure and properties of selective laser melting coating deposited on TC4 titanium alloy
Source: PLoS One. 2026 Sep 10;21(9):e0356182. doi: 10.1371/journal.pone.0356182 (PMC13561381; doi:10.1371/journal.pone.0356182)
Supplement: Supplementary file 1 [file pone.0356182.s002.docx]

We are extremely grateful for your professional comments on our article. As you have noted, there are several issues that need to be addressed. Based on your suggestions, we have made extensive corrections to the previous draft, and the specific corrections are as follows.

Journal requirements:

When submitting your revision, we need you to address these additional requirements.

1. Please ensure that your manuscript meets PLOS ONE's style requirements, including those for file naming. The PLOS ONE style templates can be found at

<https://journals.plos.org/plosone/s/file?id=wjVg/PLOSOne_formatting_sample_main_body.pdf> and

<https://journals.plos.org/plosone/s/file?id=ba62/PLOSOne_formatting_sample_title_authors_affiliations.pdf>

**Response：**Thanks for your suggestion, we have made modifications to the manuscript format according to the format requirements of your journal.

2. Thank you for stating the following financial disclosure:

“The author thanks the financial support provided by the Jiangxi Key Laboratory of Green General Aviation Power (No. Ef202480368)、State Key Laboratory of Intelligent Manufacturing Equipment and Technology (GrantNo. IMETKF2025026) and Chengdu Aeronautic Polytechnic University (No. ZZX0624088).”

Please state what role the funders took in the study.  If the funders had no role, please state: "The funders had no role in study design, data collection and analysis, decision to publish, or preparation of the manuscript."

If this statement is not correct you must amend it as needed.

Please include this amended Role of Funder statement in your cover letter; we will change the online submission form on your behalf.

**Response：**Thanks for your suggestion, the funders had no role, we state: "The funders had no role in study design, data collection and analysis, decision to publish, or preparation of the manuscript."

3. Thank you for stating the following in the Acknowledgments Section of your manuscript:

“The author thanks the financial support provided by the Jiangxi Key Laboratory of Green General Aviation Power (No. Ef202480368)、State Key Laboratory of Intelligent Manufacturing Equipment and Technology (GrantNo. IMETKF2025026) and Chengdu Aeronautic Polytechnic University (No. ZZX0624088).”

We note that you have provided funding information that is currently declared in your Funding Statement. However, funding information should not appear in the Acknowledgments section or other areas of your manuscript. We will only publish funding information present in the Funding Statement section of the online submission form.

Please remove any funding-related text from the manuscript and let us know how you would like to update your Funding Statement. Currently, your Funding Statement reads as follows:

“The author thanks the financial support provided by the Jiangxi Key Laboratory of Green General Aviation Power (No. Ef202480368)、State Key Laboratory of Intelligent Manufacturing Equipment and Technology (GrantNo. IMETKF2025026) and Chengdu Aeronautic Polytechnic University (No. ZZX0624088).”

Please include your amended statements within your cover letter; we will change the online submission form on your behalf.

If the reviewer comments include a recommendation to cite specific previously published works, please review and evaluate these publications to determine whether they are relevant and should be cited. There is no requirement to cite these works unless the editor has indicated otherwise.

**Response：**Thanks for your suggestion, we have removed the references to funding from the acknowledgments section of the manuscript. Our statement regarding the funding is as follows: “The author thanks the financial support provided by the Jiangxi Key Laboratory of Green General Aviation Power (No. Ef202480368)、State Key Laboratory of Intelligent Manufacturing Equipment and Technology (GrantNo. IMETKF2025026) and Chengdu Aeronautic Polytechnic University (No. ZZX0624088).”

Reviewer #1:

The current review investigates the effect of laser power on the microstructure and properties of deposited AlTi coating on TC4 Titanium alloy using the selective laser melting technique. The title is attractive, and the manuscript is well written and organized. However, there are some issues to be considered as follows:
- The abstract is relatively too long (it exceeds 300 words); it should be reduced to be more concise and focused within 200 words.

**Response：**We are extremely grateful for your professional comments on our article. Based on your suggestions, we have already revised the summary section, for instance, by limiting it to 200 words or less, making it more concise, clear and highlighting the key points. And the modified parts have already been marked in red.
- The authors refer to SLM as "laser selective melting"; that expression should be replaced by "selective laser melting" throughout the manuscript text.

**Response：**Thanks for your suggestion, we have made the modifications as required. We have replaced "laser selective melting" in the manuscript with "selective laser melting". And also, mark the modified parts in red.

- The problem statement should be defined in more detail by the end of the introduction section.

**Response：**Thanks for your suggestion, we have made revisions to the end of the introduction, providing a more detailed explanation of the content of the issue and clarifying the statement of the problem. And also, mark the modified parts in red.

- The AlMgSc Powder characterization should be presented in more detail.

**Response：**Thank you for pointing this out. In Section 2, we provided a more detailed analysis of the properties of aluminum-magnesium-scandium powder. For example: The apparent density is 1.42 g/cm³, and the angle of repose is 37°. The results of the powder particle size distribution measured by the laser particle size analyzer are as follows: D_10_ = 20.5 μm, D_50_ = 36.2 μm, and D_90_ = 61.1 μm. And the modified parts have already been marked in red.

- Figure 2 should be informative and indicative; the authors should label the observations and the samples according to the applied power.

**Response：**We sincerely thanks the reviewer for the valuable suggestions. We have processed Picture 2. The marked areas in the picture show the boundary of the molten pool and the absence of a molten pool boundary at 380W, etc.

- The SEM image scale should be readable in all Figures related to microscopic analysis.

**Response：**Thank you for pointing this out. We agree with this comment. We have re-annotated the size indications of all the charts related to microscopic analysis in the manuscript, including the scanning electron microscope images (such as Figures 2, 7, and 8), to ensure they are clearly legible.

- In Figure 8, the resolution of the hardness plot should be improved, in addition to adding the standard deviation of the measurement values.

**Response：**We sincerely appreciate the valuable comments from the reviewers, as these comments helped us improve the quality of the manuscript. We processed Picture 8, enhancing its clarity and adding the standard deviation of the measurement values.

- There should be a table comparing the current study results to those obtained in the literature using the proposed technique or other techniques to justify the obtained results and analysis.

**Response：**Thanks. As suggested by the reviewer, in Section 3.3 of the manuscript, we have added the current research results and compared them with the results obtained by other technologies in the literature. Additionally, a table 4 has been included to demonstrate the rationality of the obtained results and the analysis. And it is marked in red.
- The authors should illustrate the limitations of the proposed technique, as it is limited to flat surfaces and not applicable to complex geometries.

**Response：**Thanks for your suggestion, in the concluding section of Section 3.3, we have already clarified the limitations of the proposed technology and proposed that in the future, the laser cladding method can be adopted in SLM equipment to form the protective coatings for parts with complex geometries. And it is marked in red.

- The conclusion should focus on the novelty and main contributions, in addition to the future perspective.

**Response：**Thanks very much for your comments, which are very helpful for us to improve the manuscript. We have added some new insights and key contributions of this study to the conclusion section of the manuscript, and also presented an outlook for future developments. And mark the modified parts in red.

Reviewer #2: This manuscript presents a relevant and practically valuable study in the field of surface engineering of titanium alloys for aviation applications. The authors systematically demonstrate the effect of laser power variation (350–380W) on the microstructure, phase composition, elemental distribution, and microhardness of TiAl coatings formed via Selective Laser Melting (SLM) on TC4 substrates. The key finding that 370W is the optimal power level, yielding good metallurgical bonding and a maximum hardness of 641.9 HV0.2, represents a useful contribution as a process reference baseline. However, several substantial aspects require strengthening before this manuscript is suitable for publication.
1. The authors test only four laser power levels (350W, 360W, 370W, 380W) at 10W intervals without providing adequate scientific justification for why this specific range was selected. It is not explained why powers below 350W or above 380W were not investigated. With only four data points, it is difficult to draw robust conclusions about the causal relationship between laser power and coating quality. The authors are advised to either expand the testing range or provide strong thermodynamic and metallurgical reasoning explaining why the 350–380W domain was chosen as the investigation window.

**Response：**We sincerely appreciate the valuable comments from the reviewers, as these comments helped us improve the quality of the manuscript. The selection of laser power ranging from 350W to 380W as the parameter range for this study is based on two reasons:

（1）This power range was determined based on the recommendations of the powder production manufacturers and the DMP Flex350 selective laser melting equipment manufacturing company. The technical personnel from both companies believed that under this process condition, a laser power range of 300W - 500W could potentially enable the design of the desired coating. Therefore, this study initially selected a laser power range of 350W - 380W, with the scanning speed temporarily set at 1000mm/s. Subsequent tests will involve coating designs with different power and scanning speed ranges.

（2）The direct influence of the laser energy density on the behavior of the molten pool, and the laser power and scanning speed as the key regulating parameters of the energy density, are calculated according to formula (1), directly determining the melting and solidification behavior of the molten pool, and thereby affecting the forming quality of the coating.

E = P/(s×ν×t) (1)

E: represents the laser energy density (J/mm³), P: denotes the laser power (W), t: indicates the powder layer thickness (mm), s: stands for the scanning interval (mm), and v: signifies the scanning speed (mm/s). The difference of 10W in laser power may cause changes in the quality of the formed parts.

（3）In this process, the selection of laser power ranging from 350W to 380W was based on the results obtained from a large number of references and in combination with the expectations of this experiment. Eventually, the research range of laser power for this experiment was determined. For example: Reith et al. [1] adjusted the beam power and beam speed to adapt to different scanning lengths, so as to keep the line energy, surface energy, round-trip time and lateral velocity constant during the additive manufacturing process. They used an optical microscope to test the samples prepared by this method and analyzed their lateral melt pool expansion, melt pool depth, porosity and microstructure. Gao et al. [2] in this paper used laser selective melting technology and selected three different laser powers of 340 W, 350 W, and 360 W to manufacture Ti-V-Al lightweight high-temperature shape memory alloys. Ran et al. [3] used laser selective melting (SLM) technology to prepare TA32 titanium alloy samples and studied the effects of laser power (200-400 W), scanning speed (800-1200 mm·s-1) and scanning interval (90-130 μm) on the forming quality and hardness. The results showed that: as the scanning speed increased, the surface roughness of the SLM-formed TA32 titanium alloy first decreased and then increased, and the relative density and Vickers hardness gradually decreased; as the scanning interval increased, the surface roughness of the titanium alloy first decreased and then increased, and the relative density and Vickers hardness first decreased and then increased; as the laser power increased, the surface roughness of the titanium alloy first decreased and then increased, and the relative density and Vickers hardness first increased and then decreased.

[1]Reith, M.; Breuning, C.; Franke, M.; Körner, C. Impact of the Power-Dependent Beam Diameter during Electron Beam Additive Manufacturing: A Case Study with γ-TiAl. Appl. Sci. 2022, 12, 11300. <https://doi.org/10.3390/app122111300>.

[2]Gas Yuqi. Microstructure and properties of Ti-V-Al light weight high-temperature shape memoalloys fabricated by laser additive [D]. Tianjin University of Technology,2025.DOI:10.27360/d.cnki.gtlgy.2025.000239.

[3] RAN Jiangtao，JIANGFengchun，CHEN Zhuo et al. Effect of Process Parameters on Forming Quality and Hardness of TA32 Titanium Alloy Formed by Selective Laser Melting [J]. MATERIALS FOR MECHANICAL ENGINEERING,2022,46(01):47-55+60.

2. There is a fundamental inconsistency in this manuscript: the title and main claims refer to a TiAl coating, yet the powder material used is AlMgSc (an aluminum alloy), not a TiAl powder. The TiAl intermetallic phases (TiAl, TiAl_2, Ti_2Al, TiAl_3, Ti_3Al, Ti_5Al_11) form in-situ through reactions between Al from the AlMgSc powder and Ti diffusing from the TC4 substrate. The authors must explicitly distinguish between the bulk coating (AlMgSc) and the interfacial reaction zone (TiAl intermetallics), and revise the terminology throughout the manuscript to avoid misleading readers.

**Response：**Thanks for your suggestion. This paper fabricated a TiAl protective coating on the surface of titanium alloy using AlMgSc powder. The principle is that the Al in the powder undergoes a thermochromic reaction with the titanium alloy to form a TiAl intermetallic compound. The outermost AlMgSc alloy layer of the coating does not have the function of improving the surface properties of the base titanium alloy. We have revised the relevant terms throughout the manuscript. In Section 3.1, we have particularly emphasized: The interface reaction zone is the selected titanium-aluminum intermetallic compound protective coating. This is done to avoid misleading the readers and to mark the revised content in red.

3. The authors invoke the Gibbs free energy equation (ΔG = ΔH − TΔS) to explain the phase formation mechanism at each laser power level. However, not a single numerical value of ΔG, ΔH, or ΔS is presented to support these arguments. Statements such as "ΔG < 0 and sufficient nucleation driving force" without quantitative thermodynamic calculations render these arguments highly speculative. The authors are strongly advised to perform thermodynamic calculations using CALPHAD-based software (e.g., Thermo-Calc or FactSage) or to cite quantified literature data for each reported phase to substantiate their mechanistic claims.

**Response：**Thanks for your suggestion, the authors agree with the reviewers' opinion that conducting thermodynamic calculations using software based on CALPHAD (such as Thermo-Calc or FactSage) would be beneficial. We understand this. However, due to the limitations of laboratory conditions, this test experiment cannot be carried out at present. We will pay more attention to improving the experimental prototype and will do our best to incorporate thermodynamic calculations for confirmatory analysis in subsequent experiments. We sincerely hope that you can understand our approach this time.

4. The manuscript's central claim is that the coating provides surface protection for titanium components in aviation applications, yet the only mechanical test performed is Vickers microhardness. No wear resistance (tribology), adhesion/scratch test, high-temperature oxidation resistance, or corrosion resistance data are presented. Given the claimed application in demanding aerospace engine environments, the authors are strongly advised to add at minimum quantitative adhesion testing (e.g., pull-off or scratch test) and wear resistance testing to substantiate the engineering relevance they claim.

**Response：**We are extremely grateful for your professional comments on our article. Providing data on wear resistance (tribology), adhesion/scratch testing, high-temperature oxidation resistance or corrosion resistance will be beneficial for readers. We understand this. However, due to the limitations of laboratory conditions, this test experiment cannot be conducted at present. We will pay more attention to improving the experimental prototype and will do our best to provide data on wear resistance (tribology), adhesion/scratch testing, high-temperature oxidation resistance or corrosion resistance in subsequent experiments. We sincerely hope that you can understand our approach this time.

5. Although 10 hardness measurement points per test group are mentioned, hardness values are reported as single numbers without standard deviation or confidence intervals. Similarly, coating thickness values (937.5 µm; 789.5 µm; 812.5 µm; 831.6 µm) appear to be single measurements. The number of replicate specimens per power condition is not stated. The authors must include statistical analysis (mean +/- SD) for all quantitative data and clarify the number of experimental replicates to ensure the reproducibility and statistical validity of their findings.

**Response：**We sincerely thanks the reviewer for the valuable suggestions. We processed Picture 8, enhancing its clarity and adding the standard deviation of the measurement values.

6. Table 3 presents EDS data in weight percent (Wt%), yet the entire discussion uses atomic ratios for Al/Ti (24:1; 1:1.5; 1:1.8; 4:1) without showing any transparent conversion steps. Converting from weight percent to atomic ratio depends on the atomic mass of each element and must be shown explicitly. Furthermore, the Methods section states that line scanning analysis EDS was performed to determine the distribution of Ti, Al, Mg, and Sc across the interface transition zone, yet these line scan profiles are absent from the Results section. The authors must present complete EDS line scan profiles and add an atomic percent column to Table 3.

**Response：**We were really sorry for our careless mistakes. Thank you for your reminder. In the results of the EDS analysis presented in Section 3.2, we have already converted the atomic ratios to weight percentages. The authors agree with the reviewers' opinion that the line scan curve can more clearly represent the element migration. We understand this. However, the authors believe that the purpose of the manuscript is to study the element distribution in the interface reaction zone. The study of the elements on the coating surface is not the main focus of this paper, so only the element distribution in the interface area was explored. We sincerely hope that you can understand our approach this time.
7. The data show that coating thickness decreases from 937.5 µm (350W) to 789.5 µm (360W), then increases again to 812.5 µm (370W) and 831.6 µm (380W). This non-monotonic pattern is counterintuitive, the lowest power (350W) produces the thickest coating, while higher powers yield thinner coatings. The authors provide no satisfactory mechanistic explanation for this phenomenon. Factors such as material evaporation, sputtering at higher powers, or differential melt pool penetration depth need to be explicitly discussed. It should also be clarified whether these thickness values are averages from multiple measurements or single-point measurements.

**Response：**Thanks for your suggestion, the differences in sputtering at various powers or the penetration depth of the molten pool cause variations in the thickness of the coating. We have added some discussions in Section 3.1 of the manuscript and marked them in red.

8. There is a significant labeling error in the manuscript. In Figure 2, the caption reads "(8) 350W" when it should read "(d) 380W". Additionally, the name "Yong Li" appears twice in the author list (as both the 3rd and 4th author), which is an administrative error that requires immediate correction. The authors are also advised to improve the resolution of SEM and XRD figures and ensure consistent label formatting across all figures to meet PLOS ONE publication standards.

**Response：** We were really sorry for our careless mistakes. We have corrected these minor errors, enhanced the clarity of the images, and marked them in red.

9. The authors claim that this study provides "a new method and theoretical basis for protective coating preparation", yet there is no systematic discussion of what distinguishes this work from previously published studies on similar topics. Many of the cited references (particularly references 29–52) are general SLM papers with little direct relevance to the TiAl/TC4 system. The authors are advised to conduct a more focused literature review specifically on TiAl coating systems on titanium substrates, explicitly articulate the specific contribution of this study relative to the existing body of knowledge, and replace peripheral references with literature more directly relevant to the research topic.

**Response：**Thanks for your suggestion. There are not many articles specifically focusing on the titanium-aluminum coating on the titanium substrate. Therefore, the authors referred to general SLM (Selective Laser Melting) papers, which provided some textual and conceptual assistance during the writing process, and thus these papers were cited.

Reviewer #3: Article Review
Effect of Laser Power Variation on Microstructure and Properties of Selective Laser Melting Coatings Deposited on TC4 Titanium Alloy
In this article titled “Effect of Laser Power Variation on Microstructure and Properties of Selective Laser Melting Coatings Deposited on TC4 Titanium Alloy,” the authors presented a study coating formation using laser selective melting (SLM) technology on the surface of titanium alloy, focusing on the effects of different laser powers of 350-380W on the performance of TiAl coatings formed by SLM on titanium alloy surfaces. This study seems to be a useful contribution to the literature, and it can be accepted after addressing the following minor issues:
1. The abstract should be improved to enhance flow and to provide the literature gap and the importance of this study. It should be more reader-friendly for general readers, using easy and non-technical words, and should be more self-explanatory.

**Response：**We sincerely appreciate the valuable comments from the reviewers, as these comments helped us improve the quality of the manuscript.We have made comprehensive revisions to the abstract in the manuscript, adding information about the existing literature gaps in the research as well as the significance of this study. For example: The surface properties of aviation titanium alloy directly affect its service safety and reliability, and high-performance protective coatings are essential for its practical application. However, existing studies lack clear guidance on laser power parameters for preparing high-quality TiAl composite coatings on titanium alloy substrates, leaving a research gap in optimizing coating quality and performance. To fill this gap, this work used selective laser melting (SLM) to fabricate TiAl composite coatings on titanium alloy substrates, focusing on the influence of laser power (350–380 W) on coating microstructure, phase composition, elemental distribution and microhardness. And it is marked in red.

2. The Keywords should be revised to include more relevant keywords and should not include abbreviations only to improve the searchability of the article. For better Search Engine Optimization and searchability, more focused keywords should be included while keeping the abbreviations if necessary.

**Response：**We are extremely grateful for your professional comments on our article. Based on your suggestions,We have made revisions and additions to the Keywords in the manuscript. We have changed the abbreviations to their full names and added other Keywords. And mark the modified parts in red.

3. Some references are incomplete; please recheck to include all information.

**Response：**Thank you for pointing this out. I made some modifications to some incomplete reference materials and marked them in red.
4. The English language flow in this manuscript should be improved. It should also be more detailed.

**Response：**We think this is an excellent suggestion. We made every effort to improve the manuscript and made some revisions to it. These changes will not affect the content and structure of the paper. Here, we did not list the alterations, but they are marked in red in the revised document. We also enriched the corresponding content to make it more detailed.

5. The relevance of process, tool and equipment and the variables and reasoning for specific parameters used is not mentioned in detail.

**Response：**Thanks for your suggestion. In Section 2 of our manuscript, we have added some related information about the processes, tools and equipment, including some reasons for choosing the various parameter sizes in the experiments. For example:

(1)The apparent density is 1.42 g/cm³, and the angle of repose is 37°. The results of the powder particle size distribution measured by the laser particle size analyzer are as follows: D10 = 20.5 μm, D50 = 36.2 μm, and D90 = 61.1 μm;

(2)This fixation method is closely related to the printing process and the tools and equipment, and directly affects the forming accuracy of the coating and the quality of the interface bonding. The fixation of the substrate is the prerequisite for layer-by-layer melting and accumulation, which can prevent the coating from shifting and ensure uniform thickness;

(3)This parameter is determined through reasoning based on the test purpose, equipment performance, and subsequent detection requirements. A too small size will affect the subsequent performance detection, while a too large size will increase the consumption of materials and time and is prone to causing defects. This size is suitable for the printing accuracy of the equipment, can precisely achieve the coating thickness, ensure an appropriate contact area of the substrate, and provide suitable samples for subsequent performance detection.

And some explanations were given for choosing this laser power range.

The selection of laser power ranging from 350W to 380W as the parameter range for this study is based on two reasons:

（1）This power range was determined based on the recommendations of the powder production manufacturers and the DMP Flex350 selective laser melting equipment manufacturing company. The technical personnel from both companies believed that under this process condition, a laser power range of 300W - 500W could potentially enable the design of the desired coating. Therefore, this study initially selected a laser power range of 350W - 380W, with the scanning speed temporarily set at 1000mm/s. Subsequent tests will involve coating designs with different power and scanning speed ranges.

（2）The direct influence of the laser energy density on the behavior of the molten pool, and the laser power and scanning speed as the key regulating parameters of the energy density, are calculated according to formula (1), directly determining the melting and solidification behavior of the molten pool, and thereby affecting the forming quality of the coating.

E = P/(s×ν×t) (1)

E: represents the laser energy density (J/mm³), P: denotes the laser power (W), t: indicates the powder layer thickness (mm), s: stands for the scanning interval (mm), and v: signifies the scanning speed (mm/s). The difference of 10W in laser power may cause changes in the quality of the formed parts.

（3）In this process, the selection of laser power ranging from 350W to 380W was based on the results obtained from a large number of references and in combination with the expectations of this experiment. Eventually, the research range of laser power for this experiment was determined. For example: Reith et al. [1] adjusted the beam power and beam speed to adapt to different scanning lengths, so as to keep the line energy, surface energy, round-trip time and lateral velocity constant during the additive manufacturing process. They used an optical microscope to test the samples prepared by this method and analyzed their lateral melt pool expansion, melt pool depth, porosity and microstructure. Gao et al. [2] in this paper used laser selective melting technology and selected three different laser powers of 340 W, 350 W, and 360 W to manufacture Ti-V-Al lightweight high-temperature shape memory alloys. Ran et al. [3] used laser selective melting (SLM) technology to prepare TA32 titanium alloy samples and studied the effects of laser power (200-400 W), scanning speed (800-1200 mm·s-1) and scanning interval (90-130 μm) on the forming quality and hardness. The results showed that: as the scanning speed increased, the surface roughness of the SLM-formed TA32 titanium alloy first decreased and then increased, and the relative density and Vickers hardness gradually decreased; as the scanning interval increased, the surface roughness of the titanium alloy first decreased and then increased, and the relative density and Vickers hardness first decreased and then increased; as the laser power increased, the surface roughness of the titanium alloy first decreased and then increased, and the relative density and Vickers hardness first increased and then decreased.

[1]Reith, M.; Breuning, C.; Franke, M.; Körner, C. Impact of the Power-Dependent Beam Diameter during Electron Beam Additive Manufacturing: A Case Study with γ-TiAl. Appl. Sci. 2022, 12, 11300. <https://doi.org/10.3390/app122111300>.

[2]Gas Yuqi. Microstructure and properties of Ti-V-Al light weight high-temperature shape memoalloys fabricated by laser additive [D]. Tianjin University of Technology,2025.DOI:10.27360/d.cnki.gtlgy.2025.000239.

[3] RAN Jiangtao，JIANGFengchun，CHEN Zhuo et al. Effect of Process Parameters on Forming Quality and Hardness of TA32 Titanium Alloy Formed by Selective Laser Melting [J]. MATERIALS FOR MECHANICAL ENGINEERING,2022,46(01):47-55+60.

6. Coherent terminologies should be used, like figure or fig, throughout the whole manuscript.

**Response：**Thanks for your suggestion, we have already uniformly modified the pictures in the manuscript to “Fig”.
7. The motivation behind this study should be included with more detail, and it should be in proper flow, introducing the gap and research question.

**Response：**We sincerely thanks the reviewer for the valuable suggestions. We have made supplementary additions to the abstract, introduction and the ending of Section 3.3 in the manuscript, including the motivation behind the research, the differences between the techniques, and the research questions. And mark the modified parts in red.

8. The suppositions and boundary conditions should be explicitly declared.

**Response：**Thanks for your suggestion, we provide explanations based on our assumptions and boundary conditions.

Reviewer #4: This manuscript investigates the effect of laser power (350–380 W) on the microstructure and mechanical properties of coatings fabricated on TC4 titanium alloy via selective laser melting (SLM). The authors employ SEM, EDS, XRD, and microhardness testing to characterize coating morphology, phase composition, elemental distribution, and hardness evolution. The results show that laser power significantly affects coating quality: low power (350–360 W) leads to insufficient melting and severe cracking, an intermediate power (370 W) produces optimal metallurgical bonding and the highest hardness (~642 HV), and high power (380 W) causes overheating and renewed cracking. The study is experimentally straightforward and addresses a practically relevant topic, and the observed trends are generally consistent and reasonable. However, the work is largely incremental with limited novelty, the mechanistic discussion lacks quantitative support, the material system description is inconsistent (TiAl vs. AlMgSc), and the overall rigor and presentation quality are insufficient.
Recommendation: Major revision
Major issues:
(a) Lack of novelty: The influence of laser power on SLM microstructure and properties is already well established. The manuscript mainly confirms known trends without providing new insights or deeper analysis. The authors should better justify the novelty and significance of this work.

**Response：**We sincerely appreciate the valuable comments from the reviewers, as these comments helped us improve the quality of the manuscript. In the introduction, we have added some new and important aspects of this work, such as: Currently, the surface protective coatings used in the aerospace field during SLM forming have unclear effects of different laser parameters on the forming mechanism of TiAl coatings. Compared with many current coating forming technologies, the laser selective melting coating is generated and cooled within the atmosphere protection system of the equipment. This can completely prevent the formation of brittle oxides and suppress their thermal cracking behavior.The research in this paper can fill this gap, provide theoretical basis for the forming of aerospace titanium alloy surface protective coatings, offer practical technical support for improving the surface properties of titanium alloys, and also provide a new idea and method for the design and analysis of TiAl coatings. The revised content has been marked in red by us.

(b) Confusion in material system: The manuscript refers to “TiAl coatings,” while the feedstock material is AlMgSc powder. This inconsistency appears in the Abstract, Introduction, and Results (especially Section 3.2), and creates confusion regarding the coating composition and phase evolution. This issue must be clarified throughout the manuscript.

**Response：**Thanks for your suggestion. This paper fabricated a TiAl protective coating on the surface of titanium alloy using AlMgSc powder. The principle is that the Al in the powder undergoes a thermochromic reaction with the titanium alloy to form a TiAl intermetallic compound. The outermost AlMgSc alloy layer of the coating does not have the function of improving the surface properties of the base titanium alloy. We have revised the relevant terms throughout the manuscript. In Section 3.1, we have particularly emphasized: The interface reaction zone is the selected titanium-aluminum intermetallic compound protective coating. This is done to avoid misleading the readers and to mark the revised content in red.

(c) Weak mechanistic analysis: The discussion relies heavily on qualitative thermodynamic arguments (e.g., Gibbs free energy), but no quantitative calculations, phase diagram analysis, or supporting data are provided. The explanation remains speculative and should be strengthened or simplified.

**Response：**We sincerely thanks the reviewer for the valuable suggestions. Thank you for your suggestion. The authors agree with the viewpoints raised by the reviewers and we understand this. However, due to the limitations of the laboratory conditions, this test experiment cannot be carried out at present. We will place greater emphasis on improving the experimental prototype and will do our best to conduct thermodynamic calculations based on the CALPHAD software (such as Thermo-Calc or FactSage) in subsequent experiments. We sincerely hope that you can understand our approach this time.

(d) Insufficient statistical rigor: Microhardness results are presented without error bars, standard deviation, or repeatability analysis. The use of only 10 data points is insufficient to demonstrate reliability. Similar lack of quantification exists for microstructural features.

**Response：**Thank you for pointing this out. We processed Picture 8, enhancing its clarity and adding the standard deviation of the measurement values.

(e) Limited performance evaluation: The study focuses on microstructure and hardness only. For a coating study, additional performance metrics such as wear resistance, adhesion strength, or oxidation/corrosion behavior are expected.

**Response：**We are extremely grateful for your professional comments on our article. Providing data on wear resistance (tribology), adhesion/scratch testing, high-temperature oxidation resistance or corrosion resistance will be beneficial for readers. We understand this. However, due to the limitations of laboratory conditions, this test experiment cannot be conducted at present. We will pay more attention to improving the experimental prototype and will do our best to provide data on wear resistance (tribology), adhesion/scratch testing, high-temperature oxidation resistance or corrosion resistance in subsequent experiments. We sincerely hope that you can understand our approach this time.

(f) Overstated claims: Statements such as “provides a new method” and “important engineering application value” are not adequately supported and should be moderated.

**Response：**Thanks for your suggestion, we have already modified or deleted these expressions in the introduction.
Specific technical issues:
(a) Energy density equation (Section 3.1): The equation and parameter definitions are unclear and not fully consistent with standard notation. Please clarify symbols and units.

**Response：**Thanks for your suggestion, we have modified the formula.
(b) Phase identification (Section 3.2): Multiple phases are reported without detailed peak indexing or reference patterns, making the identification unclear.

**Response：**Thanks for your suggestion, we have made the necessary revisions to it.
(c) EDS results (Section 3.2, Fig. 7, Table 3): Reported atomic ratios (e.g., Al/Ti = 24:1) appear extreme. The authors should clarify whether these values are averaged and discuss measurement uncertainty.

**Response：**We were really sorry for our careless mistakes. Thank you for your reminder. In the results of the EDS analysis presented in Section 3.2, we have already converted the atomic ratios to weight percentages. The authors agree with the reviewers' opinion that the line scan curve can more clearly represent the element migration. We understand this. However, the authors believe that the purpose of the manuscript is to study the element distribution in the interface reaction zone. The study of the elements on the coating surface is not the main focus of this paper, so only the element distribution in the interface area was explored. We sincerely hope that you can understand our approach this time.

(d) Hardness discussion (Section 3.3): The explanation linking hardness to “binding energy of intermetallic compounds” is vague and lacks quantitative support.

**Response：**We sincerely appreciate the valuable comments from the reviewers, as these comments helped us improve the quality of the manuscript. We processed Picture 8, and adding the standard deviation of the measurement values.

Formatting issues:
(a) Figure 2 caption: “(8) 350W” appears incorrect and should likely be “(d) 380W”.

**Response：**We were really sorry for our careless mistakes. We have corrected these minor errors, and marked them in red.
(b) Inconsistent figure labeling across the manuscript.

**Response：**Thanks for your suggestion, We standardized the numerical annotations in the manuscript, for example:we have already uniformly modified the pictures in the manuscript to “Fig”.

(c) Table 1 formatting is unclear, with misaligned columns.

**Response：**Thank you for pointing this out. We have made modifications to Table 1.

(d) Table 3 lacks clear explanation of sample numbering (1–4).

**Response：**Thanks for your suggestion, We have added labels to the numbers in Table 3.
(e) Inconsistent unit formatting (e.g., µm vs. um, spacing issues, HV0.2 notation).

**Response：**Thank you for pointing this out. We have standardized the units in the manuscript to µm.
(f) Repetition of similar explanations across Sections 3.1–3.3.

**Response：**We have removed or modified the similar contents between Section 3.1 and Section 3.3. The modified parts are marked in red.

Language and grammar issues (selected examples):
(a) Abstract: “the phases of Ti5Al11 and Al2Sc are added on the basis of 360W” → unclear and grammatically incorrect.

**Response：**Thanks for your suggestion, we have made the necessary revisions in the abstract.
(b) Introduction: “demonstrate a wide range of applications” → should be simplified to “are widely used.”

**Response：**Thank you for pointing this out. We have made the following modification in the introduction: "demonstrate a wide range of applications" → be simplified to "are widely used."

(c) Section 3.1: Several sentences are overly long and difficult to follow.

**Response：**Thanks for your suggestion, we have made some revisions to some of the sentences in Section 3.1 and marked them in red.

(d) Section 3.2: Thermodynamic discussion contains grammatical errors and inconsistent tense usage.

**Response：**We sincerely thanks the reviewer for the valuable suggestions. We have made some revisions to some of the sentences in Section 3.2 and marked them in red.

(e) General issues: Missing articles, singular/plural inconsistencies, and repetitive phrasing (e.g., “at this point,” “at the same time”).

**Response：**Thanks for your suggestion, We have made some revisions to certain expressions in the manuscript, (e.g., "at this point", "at the same time").
(f) The manuscript requires substantial English editing for clarity and readability.

**Response：**We made every effort to improve the manuscript and made some revisions to it. These changes will not affect the content and structure of the paper. Here, we did not list the alterations, but they are marked in red in the revised document. We sincerely thank the editor/ reviewer for their enthusiastic work and hope that the corrections are approved.
