# Supplementary material for: Effect of laser power variation on microstructure and properties of selective laser melting coating deposited on TC4 titanium alloy
Source: PLoS One. 2026 Sep 10;21(9):e0356182. doi: 10.1371/journal.pone.0356182 (PMC13561381; doi:10.1371/journal.pone.0356182)
Supplement: Supplementary file 2 [file pone.0356182.s003.docx]

The authors would like to thank the editors and reviewers for the careful consideration that they have given to this work. The paper has been revised taking into consideration the reviewers’ comments. As a result, the material in the paper has been clarified and improved, and hopefully it is now acceptable for publication.

1. The novelty and scientific contribution of the work should be more clearly articulated. The observed trend of insufficient energy input causing poor bonding and excessive energy causing cracking is already well established in the SLM literature. The authors should explicitly explain what new insight this study contributes beyond parameter optimization.
**Response：**Thanks very much for your comments, which are very helpful for us to improve the manuscript.In the concluding part of the introduction of the manuscript, the authors revised the main significance of this study, highlighting the novelty and scientific contribution of the research, and explaining some new insights it has brought beyond parameter optimization. And it is marked in red.

2. The manuscript still contains ambiguity regarding the material system. The feedstock material is AlMgSc powder, while the manuscript repeatedly refers to the product as a “TiAl coating.” The authors should clearly distinguish the deposited AlMgSc layer, the Ti-Al interfacial reaction zone, and the intermetallic transition region throughout the manuscript.
**Response：**We sincerely thanks the reviewer for the valuable suggestions. The authors have made modifications to the TiAl coating in the manuscript, changing it to a multi-alloy composite coating or a composite coating. In Section 3.1, the analysis of the coating structure proposed that: the outer layer of the coating is the deposited AlMgSc layer, and the interface between the coating and the substrate is the Ti-Al reaction zone and the intermetallic compound transformation zone.

3. The mechanistic discussion remains largely qualitative. The repeated use of Gibbs free energy arguments without quantitative thermodynamic calculations, phase diagram analysis, or CALPHAD support weakens the scientific rigor of the discussion. At minimum, the speculative nature of these interpretations should be explicitly acknowledged and moderated.
**Response：**Thank you for pointing this out. We agree with this comment. I have revised the analysis of XRD in Section 3.2. I have reduced the discussion of the Gibbs free energy and acknowledged and moderately limited the speculative nature of these explanations. And explain the reasons for the formation of the new phase from the perspective of thermodynamic analysis. Marked in red.

4. The manuscript claims protective coating functionality for aerospace applications, yet no wear resistance, oxidation resistance, corrosion resistance, or adhesion testing is presented. Hardness alone is insufficient to demonstrate coating performance. The engineering claims should therefore be moderated unless additional functional testing is included.
**Response：**We sincerely appreciate the valuable comments from the reviewers, as these comments helped us improve the quality of the manuscript. We would like to express our gratitude to the reviewers for their valuable suggestions. Due to the disassembly of the samples and the return of the leased testing equipment, we are unable to provide the test results for wear resistance, oxidation resistance, corrosion resistance or adhesion. In accordance with the reviewers' suggestions, we have appropriately revised the engineering statement at the end of the introduction section of the manuscript. For example:“providing new analytical ideas and technical support for the improvement of titanium alloy surface properties and the design of coatings, but also provides reliable theoretical and experimental basis for the additive manufacturing preparation of protective coatings for aerospace titanium alloys.” At the same time, the expressions related to the engineering aspect in the conclusion part of the abstract were revised.

5. The statistical rigor should be improved further. The manuscript now includes standard deviation values for hardness measurements, which is appreciated; however, the number of independently fabricated samples per condition remains unclear. The authors should clarify the repeatability of the experiments and provide statistical treatment for coating thickness measurements as well.
**Response：**Thanks for your suggestion. In the final part of the second chapter of the manuscript, the authors demonstrate the reproducibility of the experiment. For instance, in this experiment, three parallel samples were independently prepared for each laser power condition, ensuring the reliability and repeatability of the experimental data. Meanwhile, in Section 3.1 of the manuscript, we have added a statistical analysis of the measurement results of the coating thickness. For example:For each laser power group, three independent parallel samples were prepared. Each sample was cut to prepare cross-sectional metallographic specimens. For each sample section, three observation areas were selected at equal intervals, and 5 coating thickness readings were randomly collected from each area. The average coating thickness value of each group was calculated as the final coating thickness value.

6. The literature review should be revised to focus more specifically on TiAl intermetallic coatings on titanium substrates and related interfacial reaction systems. Many currently cited references are generic SLM studies with limited direct relevance to the present material system.
**Response:**Thanks. The authors have removed some of the commonly cited references from the SLM research, such as those in references [24]-[33] and [35], [36]、[39]、[41]、[42], etc. And the labels of other references in the text have been revised. The modified parts are marked in red.
Minor comments:

1. The manuscript still contains several long and grammatically awkward sentences, particularly in Sections 3.1 and 3.2. Additional English editing is recommended.
**Response：**Thank you for pointing this out. The authors have refined the long sentences and those with improper grammar in the manuscript, especially in Sections 3.1 and 3.2, and marked them in red.

2. Figure quality has improved, but several SEM images still require clearer contrast and labeling for publication quality.
**Response：**Thanks for your suggestion. We have made modifications to the pictures in the manuscript. We have re-drawn the annotations for pictures 2 and 3, and improved the clarity of pictures 7 and 8. The pictures in the entire manuscript are much clearer.

3. The discussion of the non-monotonic coating thickness trend should be strengthened further with a more detailed explanation of melt pool dynamics and possible evaporation or recoil pressure effects.
**Response：**Thanks for your suggestion. In Section 3.2 of the manuscript, we have added an additional section in the coating SEM analysis, focusing on discussing the possible reasons for the non-monotonic coating thickness trend, providing a detailed explanation of the molten pool dynamics and the possible effects of evaporation or recoil pressure. Marked in red.

4. The terminology describing the coating should be made fully consistent throughout the manuscript.

**Response：**We sincerely thanks the reviewer for the valuable suggestions. We have standardized and unified the terms used to describe the coating throughout the entire manuscript. We have also revised the previous TiAl coating, for example: composite coating.
