# Supplementary material for: Effect of laser power variation on microstructure and properties of selective laser melting coating deposited on TC4 titanium alloy
Source: PLoS One. 2026 Sep 10;21(9):e0356182. doi: 10.1371/journal.pone.0356182 (PMC13561381; doi:10.1371/journal.pone.0356182)
Supplement: Supplementary file 3 [file pone.0356182.s004.docx]

Dear Editor,

We submit herein the revised manuscript “Effect of Laser Power Variation on Microstructure and Properties of Selective Laser Melting Coating Deposited on TC4 Titanium Alloy”, authored by Wei Xu, Gaojian You , Yong Li,Bo Tao , Xiang Li, Guoyang Zhao.

We extend our deepest appreciation to the editor and reviewers for their careful and constructive assessment of this work. We have thoroughly revised the manuscript in full response to all reviewer suggestions. Reviewers’ comments are italicized and numbered sequentially, our detailed replies are shown in blue font, and all revised text in the manuscript is highlighted red.

These valuable suggestions have greatly enhanced the integrity, clarity and scientific quality of our paper. We firmly believe the revised version satisfies the journal’s publication requirements and kindly ask for your approval for publication.

***Journal Requirements:***

*1.If the reviewer comments include a recommendation to cite specific previously published works, please review and evaluate these publications to determine whether they are relevant and should be cited. There is no requirement to cite these works unless the editor has indicated otherwise.* 
**Response：**Thanks very much for your comments, which are very helpful for us to improve the manuscript. The reviewers' comments did not mention the suggestion to cite specific published literature, and the authors themselves will not cite any specific published works.

*2. Please review your reference list to ensure that it is complete and correct. If you have cited papers that have been retracted, please include the rationale for doing so in the manuscript text, or remove these references and replace them with relevant current references. Any changes to the reference list should be mentioned in the rebuttal letter that accompanies your revised manuscript. If you need to cite a retracted article, indicate the article’s retracted status in the References list and also include a citation and full reference for the retraction notice.*

**Response：**Thank you for pointing this out. We agree with this comment. After reviewing the references, we found that reference [9] has been retracted, so it has been removed and replaced with the most recent relevant reference.

***Reviewer #1:*** *The revised manuscript is significantly improved. The review comments and recommendations have been addressed. However, some issues still need to be considered as follows:
1. In Section 2, the powder material name should be clearly stated before presenting the powder characteristics such as particle size distribution. Also, it is very crucial to illustrate the powder morphology and particle shape.*

**Response：**Thanks very much for your comments, which are very helpful for us to improve the manuscript. In the initial part of Section 2 of the manuscript, the authors added Figure 1. Figure 1 (a) shows the surface morphology of AlMgSc particles observed by the Thermo Scientific Quattro scanning electron microscope (SEM), clearly presenting the morphology and shape of the powder, and the AlMgSc matrix powder maintains a high degree of sphericity. Figure 1 (b) presents the powder particle size distribution results measured by the laser particle size analyzer, introducing the powder characteristics (such as particle size distribution).

*2.In subsection 3.2 related to phase analysis; the manuscript text should illustrate more analysis and discussion for the data presented in Table 3 related to the significant variation of elements' wt% of the EDS results throughout the manuscript text, and the discussion should be supported by relevant references.*

**Response：**Thank you for pointing this out. We agree with this comment. In Section 3.2, the authors further analyzed and discussed the significant changes in the data presented in Figure 7 (now renamed as Figure 9) and Table 3. They analyzed the changes in the mass percentages of each element in the electron energy spectrum (EDS) results, and also added appropriate references. The modified content is marked in red.

*3. The directions related to the building direction should be labelled on all Figures containing SEM and microscopic images.*

**Response：**We sincerely thanks the reviewer for the valuable suggestions. The authors have added the building directions to all the figures in the manuscript that contain scanning electron microscopy and microscopic images. In Section 3.1, the meaning of the abbreviation "BD" is specifically explained, for example, "building direction (BD) refers to the construction direction."

*4.The references of the presented data in Figure 4 should be cited and illustrated in a separate column in the Table.*

**Response：**Thanks for your suggestion, the data in Picture 4 are the data used in this experiment. The purpose is to show the influence of different laser powers on the coating quality in this experiment. No data from other references were used or cited in the picture.

***Reviewer #4:*** *The revised manuscript presents a systematic experimental investigation of the effect of laser power on the microstructure, phase evolution, elemental distribution, and microhardness of selective laser melting (SLM) coatings deposited on TC4 titanium alloy. The experimental design is straightforward, and the combination of SEM, EDS, XRD, and hardness measurements provides a comprehensive characterization of the coatings. The manuscript is generally well organized, and the experimental results consistently demonstrate that a laser power of 370 W produces the most favorable coating quality with improved metallurgical bonding and the highest microhardness.

The current version has been substantially improved in terms of experimental description, statistical information, and presentation of the results. The distinction between the deposited layer and the interfacial reaction zone is much clearer, the experimental repeatability is better documented, and the discussion has been strengthened with additional analysis of coating thickness evolution and microstructural development. Overall, the study is technically sound and provides useful experimental data for understanding the influence of laser power on this specific coating system.

I only have a few minor comments that should be addressed before publication.

1.The English writing has improved, but the manuscript would still benefit from careful language editing. Several sentences remain grammatically awkward or unnecessarily long, particularly in the Introduction and Discussion sections. A final proofreading by a fluent English speaker is recommended to improve readability.*
**Response：** We sincerely appreciate the valuable comments from the reviewers, as these comments helped us improve the quality of the manuscript. The authors have thoroughly reviewed the manuscript in response to the reviewers' comments, making every effort to improve the content and completing numerous targeted revisions. All the modifications made this time are aimed at addressing issues such as grammatical incoherence or unnecessary length in some sentences, with a focus on the introduction and discussion sections. The adjusted content is marked in red font throughout the revised manuscript. We will not list each item separately here. We sincerely thank the editor and all the reviewers for spending their valuable time carefully reviewing this article. We also greatly appreciate the professional guidance provided by all of you. We earnestly request that the editor and reviewers approve these revisions.

*2.The manuscript occasionally overstates the significance of its findings. Expressions such as "fills the existing research gap," "forms a new understanding," and "provides theoretical support" should be moderated. The work represents a systematic experimental study of laser power optimization for a particular material system, and the conclusions should reflect this scope more appropriately.*
**Response：**We sincerely appreciate your objective, fair, and meticulous review comments. The authors removed "fills the existing research gap" at the end of the abstract and replaced it with "This research provides certain technical references and theoretical support for the preparation of TiAl coatings on the surface of aerospace titanium alloys."

The authors removed the sentence "This study breaks away from the traditional framework of single-component coating research" in the conclusion section, and changed the following: "Provide new theoretical support and practical references for the parameter optimization and performance regulation of titanium alloy surface strengthening processes." to: "It provides certain theoretical support and reference for the parameter optimization and performance regulation of the TiAl coating on the titanium alloy surface." *3.Some mechanistic explanations remain largely qualitative. Discussions involving Gibbs free energy, recoil pressure, keyhole transition, and phase evolution are reasonable interpretations but are not supported by quantitative thermodynamic calculations or numerical simulations. The corresponding statements should therefore be presented as proposed mechanisms rather than definitive conclusions.*
**Response：**Thank you for your reminder. In Section 3.1 of the manuscript, the authors added some explanations, stating that the corresponding statements represent the proposed mechanism, such as: "The possible reasons might be,...". Some of the proposed mechanisms in the manuscript were made based on explanations derived from other referenced literature. In Section 3.2, replace: "The thermodynamic calculation showed that at this time," with "According to thermodynamic calculations,". And mark the modified parts in red.

*4.Although the terminology has been improved, some inconsistency remains in the description of the coating. Terms such as "TiAl coating," "composite coating," and "protective coating" are still used interchangeably in several sections. Using consistent terminology throughout the manuscript would improve clarity.*
**Response：**Thank you for your reminder. The authors have uniformly referred to "TiAl coating," "composite coating," and "protective coating" as "TiAl coating."

*5.Finally, several minor typographical and formatting issues remain, including duplicated words in the Abstract, occasional spacing inconsistencies, and minor formatting problems for units and symbols. These should be corrected during the final proofreading stage.*
**Response：**Thanks for your suggestion, the authors have carefully checked the minor layout and formatting issues in the manuscript, corrected the minor formatting problems of units and symbols, and marked them in red.

*Overall, the manuscript is technically sound, the experimental methodology is appropriate, and the conclusions are generally supported by the presented results. The remaining issues are minor editorial revisions that do not require additional experiments. Therefore, I recommend **acceptance after minor revision**.*

We again extend our deepest appreciation to the editorial team and reviewers for your careful and professional guidance. All revisions in this manuscript have been completed rigorously following your detailed suggestions. Please do not hesitate to share any further revisions or adjustments if the paper still requires polishing. We eagerly await your feedback.
