# Supplementary material for: Effect of laser power variation on microstructure and properties of selective laser melting coating deposited on TC4 titanium alloy
Source: PLoS One. 2026 Sep 10;21(9):e0356182. doi: 10.1371/journal.pone.0356182 (PMC13561381; doi:10.1371/journal.pone.0356182)
Supplement: Peer review history

# Peer review: Decision Letter 0

**DOI:** 10.1371/journal.pone.0356182.r001

**Authors:** Hari Murthy

25 Mar 2026

Dear Dr. Yong,

Thank you for submitting your manuscript to PLOS ONE. After careful consideration, we feel that it has merit but does not fully meet PLOS ONE’s publication criteria as it currently stands. Therefore, we invite you to submit a revised version of the manuscript that addresses the points raised during the review process.

==============================

Please submit your revised manuscript by May 09 2026 11:59PM. If you will need more time than this to complete your revisions, please reply to this message or contact the journal office at plosone@plos.org. When you're ready to submit your revision, log on to https://www.editorialmanager.com/pone/ and select the 'Submissions Needing Revision' folder to locate your manuscript file.

- A letter that responds to each point raised by the academic editor and reviewer(s). You should upload this letter as a separate file labeled 'Response to Reviewers'.
- A marked-up copy of your manuscript that highlights changes made to the original version. You should upload this as a separate file labeled 'Revised Manuscript with Track Changes'.
- An unmarked version of your revised paper without tracked changes. You should upload this as a separate file labeled 'Manuscript'.

If you would like to make changes to your financial disclosure, please include your updated statement in your cover letter. Guidelines for resubmitting your figure files are available below the reviewer comments at the end of this letter.

If applicable, we recommend that you deposit your laboratory protocols in protocols.io to enhance the reproducibility of your results. Protocols.io assigns your protocol its own identifier (DOI) so that it can be cited independently in the future. For instructions see: https://journals.plos.org/plosone/s/submission-guidelines#loc-laboratory-protocols. Additionally, PLOS ONE offers an option for publishing peer-reviewed Lab Protocol articles, which describe protocols hosted on protocols.io. Read more information on sharing protocols at https://plos.org/protocols?utm_medium=editorial-email&utm_source=authorletters&utm_campaign=protocols.

As the corresponding author, your ORCID iD is verified in the submission system and will appear in the published article. PLOS supports the use of ORCID, and we encourage all coauthors to register for an ORCID iD and use it as well. Please encourage your coauthors to verify their ORCID iD within the submission system before final acceptance, as unverified ORCID iDs will not appear in the published article. *Only*  the individual author can complete the verification step; PLOS staff *cannot*  verify ORCID iDs on behalf of authors.

We look forward to receiving your revised manuscript.

Kind regards,

Hari Murthy, Ph.D.

Academic Editor

PLOS One

Journal requirements:

When submitting your revision, we need you to address these additional requirements.

1. Please ensure that your manuscript meets PLOS ONE's style requirements, including those for file naming. The PLOS ONE style templates can be found at

https://journals.plos.org/plosone/s/file?id=wjVg/PLOSOne_formatting_sample_main_body.pdf and

https://journals.plos.org/plosone/s/file?id=ba62/PLOSOne_formatting_sample_title_authors_affiliations.pdf

2. Thank you for stating the following financial disclosure:

“The author thanks the financial support provided by the Jiangxi Key Laboratory of Green General Aviation Power (No. Ef202480368)、State Key Laboratory of Intelligent Manufacturing Equipment and Technology (GrantNo. IMETKF2025026) and Chengdu Aeronautic Polytechnic University (No. ZZX0624088).”

Please state what role the funders took in the study.  If the funders had no role, please state: "The funders had no role in study design, data collection and analysis, decision to publish, or preparation of the manuscript."

If this statement is not correct you must amend it as needed.

Please include this amended Role of Funder statement in your cover letter; we will change the online submission form on your behalf.

3. Thank you for stating the following in the Acknowledgments Section of your manuscript:

“The author thanks the financial support provided by the Jiangxi Key Laboratory of Green General Aviation Power (No. Ef202480368)、State Key Laboratory of Intelligent Manufacturing Equipment and Technology (GrantNo. IMETKF2025026) and Chengdu Aeronautic Polytechnic University (No. ZZX0624088).”

We note that you have provided funding information that is currently declared in your Funding Statement. However, funding information should not appear in the Acknowledgments section or other areas of your manuscript. We will only publish funding information present in the Funding Statement section of the online submission form.

Please remove any funding-related text from the manuscript and let us know how you would like to update your Funding Statement. Currently, your Funding Statement reads as follows:

“The author thanks the financial support provided by the Jiangxi Key Laboratory of Green General Aviation Power (No. Ef202480368)、State Key Laboratory of Intelligent Manufacturing Equipment and Technology (GrantNo. IMETKF2025026) and Chengdu Aeronautic Polytechnic University (No. ZZX0624088).”

Please include your amended statements within your cover letter; we will change the online submission form on your behalf.

If the reviewer comments include a recommendation to cite specific previously published works, please review and evaluate these publications to determine whether they are relevant and should be cited. There is no requirement to cite these works unless the editor has indicated otherwise.

[Note: HTML markup is below. Please do not edit.]

Reviewers' comments:

Reviewer's Responses to Questions

**Comments to the Author**

1. Is the manuscript technically sound, and do the data support the conclusions?

Reviewer #1: Yes

Reviewer #2: Partly

Reviewer #3: Yes

Reviewer #4: Yes

**********

2. Has the statistical analysis been performed appropriately and rigorously?

Reviewer #1: Yes

Reviewer #2: No

Reviewer #3: Yes

Reviewer #4: Yes

**********

3. Have the authors made all data underlying the findings in their manuscript fully available?

The PLOS Data policy

Reviewer #1: Yes

Reviewer #2: Yes

Reviewer #3: Yes

Reviewer #4: Yes

**********

4. Is the manuscript presented in an intelligible fashion and written in standard English?

Reviewer #1: Yes

Reviewer #2: No

Reviewer #3: Yes

Reviewer #4: No

**********

Reviewer #1: The current review investigates the effect of laser power on the microstructure and properties of deposited AlTi coating on TC4 Titanium alloy using the selective laser melting technique. The title is attractive, and the manuscript is well written and organized. However, there are some issues to be considered as follows:

- The abstract is relatively too long (it exceeds 300 words); it should be reduced to be more concise and focused within 200 words.

- The authors refer to SLM as "laser selective melting"; that expression should be replaced by "selective laser melting" throughout the manuscript text.

- The problem statement should be defined in more detail by the end of the introduction section.

- The AlMgSc Powder characterization should be presented in more detail.

- Figure 2 should be informative and indicative; the authors should label the observations and the samples according to the applied power.

- The SEM image scale should be readable in all Figures related to microscopic analysis.

- In Figure 8, the resolution of the hardness plot should be improved, in addition to adding the standard deviation of the measurement values.

- There should be a table comparing the current study results to those obtained in the literature using the proposed technique or other techniques to justify the obtained results and analysis.

- The authors should illustrate the limitations of the proposed technique, as it is limited to flat surfaces and not applicable to complex geometries.

- The conclusion should focus on the novelty and main contributions, in addition to the future perspective.

- Another review round is highly recommended after considering the above comments and recommendations.

Reviewer #2: This manuscript presents a relevant and practically valuable study in the field of surface engineering of titanium alloys for aviation applications. The authors systematically demonstrate the effect of laser power variation (350–380W) on the microstructure, phase composition, elemental distribution, and microhardness of TiAl coatings formed via Selective Laser Melting (SLM) on TC4 substrates. The key finding that 370W is the optimal power level, yielding good metallurgical bonding and a maximum hardness of 641.9 HV0.2, represents a useful contribution as a process reference baseline. However, several substantial aspects require strengthening before this manuscript is suitable for publication.

1. The authors test only four laser power levels (350W, 360W, 370W, 380W) at 10W intervals without providing adequate scientific justification for why this specific range was selected. It is not explained why powers below 350W or above 380W were not investigated. With only four data points, it is difficult to draw robust conclusions about the causal relationship between laser power and coating quality. The authors are advised to either expand the testing range or provide strong thermodynamic and metallurgical reasoning explaining why the 350–380W domain was chosen as the investigation window.

2. There is a fundamental inconsistency in this manuscript: the title and main claims refer to a TiAl coating, yet the powder material used is AlMgSc (an aluminum alloy), not a TiAl powder. The TiAl intermetallic phases (TiAl, TiAl_2, Ti_2Al, TiAl_3, Ti_3Al, Ti_5Al_11) form in-situ through reactions between Al from the AlMgSc powder and Ti diffusing from the TC4 substrate. The authors must explicitly distinguish between the bulk coating (AlMgSc) and the interfacial reaction zone (TiAl intermetallics), and revise the terminology throughout the manuscript to avoid misleading readers.

3. The authors invoke the Gibbs free energy equation (ΔG = ΔH − TΔS) to explain the phase formation mechanism at each laser power level. However, not a single numerical value of ΔG, ΔH, or ΔS is presented to support these arguments. Statements such as "ΔG < 0 and sufficient nucleation driving force" without quantitative thermodynamic calculations render these arguments highly speculative. The authors are strongly advised to perform thermodynamic calculations using CALPHAD-based software (e.g., Thermo-Calc or FactSage) or to cite quantified literature data for each reported phase to substantiate their mechanistic claims.

4. The manuscript's central claim is that the coating provides surface protection for titanium components in aviation applications, yet the only mechanical test performed is Vickers microhardness. No wear resistance (tribology), adhesion/scratch test, high-temperature oxidation resistance, or corrosion resistance data are presented. Given the claimed application in demanding aerospace engine environments, the authors are strongly advised to add at minimum quantitative adhesion testing (e.g., pull-off or scratch test) and wear resistance testing to substantiate the engineering relevance they claim.

5. Although 10 hardness measurement points per test group are mentioned, hardness values are reported as single numbers without standard deviation or confidence intervals. Similarly, coating thickness values (937.5 µm; 789.5 µm; 812.5 µm; 831.6 µm) appear to be single measurements. The number of replicate specimens per power condition is not stated. The authors must include statistical analysis (mean +/- SD) for all quantitative data and clarify the number of experimental replicates to ensure the reproducibility and statistical validity of their findings.

6. Table 3 presents EDS data in weight percent (Wt%), yet the entire discussion uses atomic ratios for Al/Ti (24:1; 1:1.5; 1:1.8; 4:1) without showing any transparent conversion steps. Converting from weight percent to atomic ratio depends on the atomic mass of each element and must be shown explicitly. Furthermore, the Methods section states that line scanning analysis EDS was performed to determine the distribution of Ti, Al, Mg, and Sc across the interface transition zone, yet these line scan profiles are absent from the Results section. The authors must present complete EDS line scan profiles and add an atomic percent column to Table 3.

7. The data show that coating thickness decreases from 937.5 µm (350W) to 789.5 µm (360W), then increases again to 812.5 µm (370W) and 831.6 µm (380W). This non-monotonic pattern is counterintuitive, the lowest power (350W) produces the thickest coating, while higher powers yield thinner coatings. The authors provide no satisfactory mechanistic explanation for this phenomenon. Factors such as material evaporation, sputtering at higher powers, or differential melt pool penetration depth need to be explicitly discussed. It should also be clarified whether these thickness values are averages from multiple measurements or single-point measurements.

8. There is a significant labeling error in the manuscript. In Figure 2, the caption reads "(8) 350W" when it should read "(d) 380W". Additionally, the name "Yong Li" appears twice in the author list (as both the 3rd and 4th author), which is an administrative error that requires immediate correction. The authors are also advised to improve the resolution of SEM and XRD figures and ensure consistent label formatting across all figures to meet PLOS ONE publication standards.

9. The authors claim that this study provides "a new method and theoretical basis for protective coating preparation", yet there is no systematic discussion of what distinguishes this work from previously published studies on similar topics. Many of the cited references (particularly references 29–52) are general SLM papers with little direct relevance to the TiAl/TC4 system. The authors are advised to conduct a more focused literature review specifically on TiAl coating systems on titanium substrates, explicitly articulate the specific contribution of this study relative to the existing body of knowledge, and replace peripheral references with literature more directly relevant to the research topic.

Reviewer #3: Article Review

Effect of Laser Power Variation on Microstructure and Properties of Selective Laser Melting Coatings Deposited on TC4 Titanium Alloy

In this article titled “Effect of Laser Power Variation on Microstructure and Properties of Selective Laser Melting Coatings Deposited on TC4 Titanium Alloy,” the authors presented a study coating formation using laser selective melting (SLM) technology on the surface of titanium alloy, focusing on the effects of different laser powers of 350-380W on the performance of TiAl coatings formed by SLM on titanium alloy surfaces. This study seems to be a useful contribution to the literature, and it can be accepted after addressing the following minor issues:

1. The abstract should be improved to enhance flow and to provide the literature gap and the importance of this study. It should be more reader-friendly for general readers, using easy and non-technical words, and should be more self-explanatory.

2. The Keywords should be revised to include more relevant keywords and should not include abbreviations only to improve the searchability of the article. For better Search Engine Optimization and searchability, more focused keywords should be included while keeping the abbreviations if necessary.

3. Some references are incomplete; please recheck to include all information.

4. The English language flow in this manuscript should be improved. It should also be more detailed.

5. The relevance of process, tool and equipment and the variables and reasoning for specific parameters used is not mentioned in detail.

6. Coherent terminologies should be used, like figure or fig, throughout the whole manuscript.

7. The motivation behind this study should be included with more detail, and it should be in proper flow, introducing the gap and research question.

8. The suppositions and boundary conditions should be explicitly declared.

The End

Reviewer #4: This manuscript investigates the effect of laser power (350–380 W) on the microstructure and mechanical properties of coatings fabricated on TC4 titanium alloy via selective laser melting (SLM). The authors employ SEM, EDS, XRD, and microhardness testing to characterize coating morphology, phase composition, elemental distribution, and hardness evolution. The results show that laser power significantly affects coating quality: low power (350–360 W) leads to insufficient melting and severe cracking, an intermediate power (370 W) produces optimal metallurgical bonding and the highest hardness (~642 HV), and high power (380 W) causes overheating and renewed cracking. The study is experimentally straightforward and addresses a practically relevant topic, and the observed trends are generally consistent and reasonable. However, the work is largely incremental with limited novelty, the mechanistic discussion lacks quantitative support, the material system description is inconsistent (TiAl vs. AlMgSc), and the overall rigor and presentation quality are insufficient.

Recommendation: Major revision

Major issues:

(a) Lack of novelty: The influence of laser power on SLM microstructure and properties is already well established. The manuscript mainly confirms known trends without providing new insights or deeper analysis. The authors should better justify the novelty and significance of this work.

(b) Confusion in material system: The manuscript refers to “TiAl coatings,” while the feedstock material is AlMgSc powder. This inconsistency appears in the Abstract, Introduction, and Results (especially Section 3.2), and creates confusion regarding the coating composition and phase evolution. This issue must be clarified throughout the manuscript.

(c) Weak mechanistic analysis: The discussion relies heavily on qualitative thermodynamic arguments (e.g., Gibbs free energy), but no quantitative calculations, phase diagram analysis, or supporting data are provided. The explanation remains speculative and should be strengthened or simplified.

(d) Insufficient statistical rigor: Microhardness results are presented without error bars, standard deviation, or repeatability analysis. The use of only 10 data points is insufficient to demonstrate reliability. Similar lack of quantification exists for microstructural features.

(e) Limited performance evaluation: The study focuses on microstructure and hardness only. For a coating study, additional performance metrics such as wear resistance, adhesion strength, or oxidation/corrosion behavior are expected.

(f) Overstated claims: Statements such as “provides a new method” and “important engineering application value” are not adequately supported and should be moderated.

Specific technical issues:

(a) Energy density equation (Section 3.1): The equation and parameter definitions are unclear and not fully consistent with standard notation. Please clarify symbols and units.

(b) Phase identification (Section 3.2): Multiple phases are reported without detailed peak indexing or reference patterns, making the identification unclear.

(c) EDS results (Section 3.2, Fig. 7, Table 3): Reported atomic ratios (e.g., Al/Ti = 24:1) appear extreme. The authors should clarify whether these values are averaged and discuss measurement uncertainty.

(d) Hardness discussion (Section 3.3): The explanation linking hardness to “binding energy of intermetallic compounds” is vague and lacks quantitative support.

Formatting issues:

(a) Figure 2 caption: “(8) 350W” appears incorrect and should likely be “(d) 380W”.

(b) Inconsistent figure labeling across the manuscript.

(c) Table 1 formatting is unclear, with misaligned columns.

(d) Table 3 lacks clear explanation of sample numbering (1–4).

(e) Inconsistent unit formatting (e.g., µm vs. um, spacing issues, HV0.2 notation).

(f) Repetition of similar explanations across Sections 3.1–3.3.

Language and grammar issues (selected examples):

(a) Abstract: “the phases of Ti5Al11 and Al2Sc are added on the basis of 360W” → unclear and grammatically incorrect.

(b) Introduction: “demonstrate a wide range of applications” → should be simplified to “are widely used.”

(c) Section 3.1: Several sentences are overly long and difficult to follow.

(d) Section 3.2: Thermodynamic discussion contains grammatical errors and inconsistent tense usage.

(e) General issues: Missing articles, singular/plural inconsistencies, and repetitive phrasing (e.g., “at this point,” “at the same time”).

(f) The manuscript requires substantial English editing for clarity and readability.

**********

what does this mean?). If published, this will include your full peer review and any attached files.

If you choose “no”, your identity will remain anonymous but your review may still be made public.

**Do you want your identity to be public for this peer review?**  For information about this choice, including consent withdrawal, please see our Privacy Policy

Reviewer #1: No

Reviewer #2: No

Reviewer #3: **Yes:** Ali Raza Shafqat

Reviewer #4: No

**********

[NOTE: If reviewer comments were submitted as an attachment file, they will be attached to this email and accessible via the submission site. Please log into your account, locate the manuscript record, and check for the action link "View Attachments". If this link does not appear, there are no attachment files.]

To ensure your figures meet our technical requirements, please review our figure guidelines: https://journals.plos.org/plosone/s/figures

You may also use PLOS’s free figure tool, NAAS, to help you prepare publication quality figures: https://journals.plos.org/plosone/s/figures#loc-tools-for-figure-preparation.

NAAS will assess whether your figures meet our technical requirements by comparing each figure against our figure specifications.

# Author comment: Author response to Decision Letter 1

**DOI:** 10.1371/journal.pone.0356182.r002

2 May 2026

We are extremely grateful for your professional comments on our article. As you have noted, there are several issues that need to be addressed. Based on your suggestions, we have made extensive corrections to the previous draft, and the specific corrections are as follows.

Journal requirements:

When submitting your revision, we need you to address these additional requirements.

1. Please ensure that your manuscript meets PLOS ONE's style requirements, including those for file naming. The PLOS ONE style templates can be found at

https://journals.plos.org/plosone/s/file?id=wjVg/PLOSOne_formatting_sample_main_body.pdf and

https://journals.plos.org/plosone/s/file?id=ba62/PLOSOne_formatting_sample_title_authors_affiliations.pdf

Response：Thanks for your suggestion, we have made modifications to the manuscript format according to the format requirements of your journal.

2. Thank you for stating the following financial disclosure:

“The author thanks the financial support provided by the Jiangxi Key Laboratory of Green General Aviation Power (No. Ef202480368)、State Key Laboratory of Intelligent Manufacturing Equipment and Technology (GrantNo. IMETKF2025026) and Chengdu Aeronautic Polytechnic University (No. ZZX0624088).”

Please state what role the funders took in the study. If the funders had no role, please state: "The funders had no role in study design, data collection and analysis, decision to publish, or preparation of the manuscript."

If this statement is not correct you must amend it as needed.

Please include this amended Role of Funder statement in your cover letter; we will change the online submission form on your behalf.

Response：Thanks for your suggestion, the funders had no role, we state: "The funders had no role in study design, data collection and analysis, decision to publish, or preparation of the manuscript."

3. Thank you for stating the following in the Acknowledgments Section of your manuscript:

“The author thanks the financial support provided by the Jiangxi Key Laboratory of Green General Aviation Power (No. Ef202480368)、State Key Laboratory of Intelligent Manufacturing Equipment and Technology (GrantNo. IMETKF2025026) and Chengdu Aeronautic Polytechnic University (No. ZZX0624088).”

We note that you have provided funding information that is currently declared in your Funding Statement. However, funding information should not appear in the Acknowledgments section or other areas of your manuscript. We will only publish funding information present in the Funding Statement section of the online submission form.

Please remove any funding-related text from the manuscript and let us know how you would like to update your Funding Statement. Currently, your Funding Statement reads as follows:

“The author thanks the financial support provided by the Jiangxi Key Laboratory of Green General Aviation Power (No. Ef202480368)、State Key Laboratory of Intelligent Manufacturing Equipment and Technology (GrantNo. IMETKF2025026) and Chengdu Aeronautic Polytechnic University (No. ZZX0624088).”

Please include your amended statements within your cover letter; we will change the online submission form on your behalf.

If the reviewer comments include a recommendation to cite specific previously published works, please review and evaluate these publications to determine whether they are relevant and should be cited. There is no requirement to cite these works unless the editor has indicated otherwise.

Response：Thanks for your suggestion, we have removed the references to funding from the acknowledgments section of the manuscript. Our statement regarding the funding is as follows: “The author thanks the financial support provided by the Jiangxi Key Laboratory of Green General Aviation Power (No. Ef202480368)、State Key Laboratory of Intelligent Manufacturing Equipment and Technology (GrantNo. IMETKF2025026) and Chengdu Aeronautic Polytechnic University (No. ZZX0624088).”

Reviewer #1:

The current review investigates the effect of laser power on the microstructure and properties of deposited AlTi coating on TC4 Titanium alloy using the selective laser melting technique. The title is attractive, and the manuscript is well written and organized. However, there are some issues to be considered as follows:

- The abstract is relatively too long (it exceeds 300 words); it should be reduced to be more concise and focused within 200 words.

Response：We are extremely grateful for your professional comments on our article. Based on your suggestions, we have already revised the summary section, for instance, by limiting it to 200 words or less, making it more concise, clear and highlighting the key points. And the modified parts have already been marked in red.

- The authors refer to SLM as "laser selective melting"; that expression should be replaced by "selective laser melting" throughout the manuscript text.

Response：Thanks for your suggestion, we have made the modifications as required. We have replaced "laser selective melting" in the manuscript with "selective laser melting". And also, mark the modified parts in red.

- The problem statement should be defined in more detail by the end of the introduction section.

Response：Thanks for your suggestion, we have made revisions to the end of the introduction, providing a more detailed explanation of the content of the issue and clarifying the statement of the problem. And also, mark the modified parts in red.

- The AlMgSc Powder characterization should be presented in more detail.

Response：Thank you for pointing this out. In Section 2, we provided a more detailed analysis of the properties of aluminum-magnesium-scandium powder. For example: The apparent density is 1.42 g/cm³, and the angle of repose is 37°. The results of the powder particle size distribution measured by the laser particle size analyzer are as follows: D10 = 20.5 μm, D50 = 36.2 μm, and D90 = 61.1 μm. And the modified parts have already been marked in red.

- Figure 2 should be informative and indicative; the authors should label the observations and the samples according to the applied power.

Response：We sincerely thanks the reviewer for the valuable suggestions. We have processed Picture 2. The marked areas in the picture show the boundary of the molten pool and the absence of a molten pool boundary at 380W, etc.

- The SEM image scale should be readable in all Figures related to microscopic analysis.

Response：Thank you for pointing this out. We agree with this comment. We have re-annotated the size indications of all the charts related to microscopic analysis in the manuscript, including the scanning electron microscope images (such as Figures 2, 7, and 8), to ensure they are clearly legible.

- In Figure 8, the resolution of the hardness plot should be improved, in addition to adding the standard deviation of the measurement values.

Response：We sincerely appreciate the valuable comments from the reviewers, as these comments helped us improve the quality of the manuscript. We processed Picture 8, enhancing its clarity and adding the standard deviation of the measurement values.

- There should be a table comparing the current study results to those obtained in the literature using the proposed technique or other techniques to justify the obtained results and analysis.

Response：Thanks. As suggested by the reviewer, in Section 3.3 of the manuscript, we have added the current research results and compared them with the results obtained by other technologies in the literature. Additionally, a table 4 has been included to demonstrate the rationality of the obtained results and the analysis. And it is marked in red.

- The authors should illustrate the limitations of the proposed technique, as it is limited to flat surfaces and not applicable to complex geometries.

Response：Thanks for your suggestion, in the concluding section of Section 3.3, we have already clarified the limitations of the proposed technology and proposed that in the future, the laser cladding method can be adopted in SLM equipment to form the protective coatings for parts with complex geometries. And it is marked in red.

- The conclusion should focus on the novelty and main contributions, in addition to the future perspective.

Response：Thanks very much for your comments, which are very helpful for us to improve the manuscript. We have added some new insights and key contributions of this study to the conclusion section of the manuscript, and also presented an outlook for future developments. And mark the modified parts in red.

Reviewer #2: This manuscript presents a relevant and practically valuable study in the field of surface engineering of titanium alloys for aviation applications. The authors systematically demonstrate the effect of laser power variation (350–380W) on the microstructure, phase composition, elemental distribution, and microhardness of TiAl coatings formed via Selective Laser Melting (SLM) on TC4 substrates. The key finding that 370W is the optimal power level, yielding good metallurgical bonding and a maximum hardness of 641.9 HV0.2, represents a useful contribution as a process reference baseline. However, several substantial aspects require strengthening before this manuscript is suitable for publication.

1. The authors test only four laser power levels (350W, 360W, 370W, 380W) at 10W intervals without providing adequate scientific justification for why this specific range was selected. It is not explained why powers below 350W or above 380W were not investigated. With only four data points, it is difficult to draw robust conclusions about the causal relationship between laser power and coating quality. The authors are advised to either expand the testing range or provide strong thermodynamic and metallurgical reasoning explaining why the 350–380W domain was chosen as the investigation window.

Response：We sincerely appreciate the valuable comments from the reviewers, as these comments helped us improve the quality of the manuscript. The selection of laser power ranging from 350W to 380W as the parameter range for this study is based on two reasons:

（1）This power range was determined based on the recommendations of the powder production manufacturers and the DMP Flex350 selective laser melting equipment manufacturing company. The technical personnel from both companies believed that under this process condition, a laser power range of 300W - 500W could potentially enable the design of the desired coating. Therefore, this study initially selected a laser power range of 350W - 380W, with the scanning speed temporarily set at 1000mm/s. Subsequent tests will involve coating designs with different power and scanning speed ranges.

（2）The direct influence of the laser energy density on the behavior of the molten pool, and the laser power and scanning speed as the key regulating parameters of the energy density, are calculated according to formula (1), directly determining the melting and solidification behavior of the molten pool, and thereby affecting the forming quality of the coating.

E = P/(s×ν×t) (1)

E: represents the laser energy density (J/mm³), P: denotes the laser power (W), t: indicates the powder layer thickness (mm), s: stands for the scanning interval (mm), and v: signifies the scanning speed (mm/s). The difference of 10W in laser power may cause changes in the quality of the formed parts.

（3）In this process, the selection of laser power ranging from 350W to 380W was based on the results obtained from a large number of references and in combination with the expectations of this experiment. Eventually, the research range of laser power for this experiment was determined. For example: Reith et al. [1] adjusted the beam power and beam speed to adapt to different scanning lengths, so as to keep the line energy, surface energy, round-trip time and lateral velocity constant during the additive manufacturing process. They used an optical microscope to test the samples prepared by this method and analyzed their lateral melt pool expansion, melt pool depth, porosity and microstructure. Gao et al. [2] in this paper used laser selective melting technology and selected three different laser powers of 340 W, 350 W, and 360 W to manufacture Ti-V-Al lightweight high-temperature shape memory alloys. Ran et al. [3] used laser selective melting (SLM) technology to prepare TA32 titanium alloy samples and studied the effects of laser power (200-400 W), scanning speed (800-1200 mm·s-1) and scanning interval (90-130 μm) on the forming quality and hardness. The results showed that: as the scanning speed increased, the surface roughness of the SLM-formed TA32 titanium alloy first decreased and then increased, and the relative density and Vickers hardness gradually decreased; as the scanning interval increased, the surface roughness of the titanium alloy first decreased and then increased, and the relative density and Vickers hardness first decreased and then increased; as the laser power increased, the surface roughness of the titanium alloy first decreased and then increased, and the relative density and Vickers hardness first increased and then decreased.

[1]Reith, M.; Breuning, C.; Franke, M.; Körner, C. Impact of the Power-Dependent Beam Diameter during Electron Beam Additive Manufacturing: A Case Study with γ-TiAl. Appl. Sci. 2022, 12, 11300. https://doi.org/10.3390/app122111300.

[2]Gas Yuqi. Microstructure and properties of Ti-V-Al light weight high-temperature shape memoalloys fabricated by laser additive [D]. Tianjin University of Technology,2025.DOI:10.27360/d.cnki.gtlgy.2025.000239.

[3] RAN Jiangtao，JIANGFengchun，CHEN Zhuo et al. Effect of Process Parameters on Forming Quality and Hardness of TA32 Titanium Alloy Formed by Selective Laser Melting [J]. MATERIALS FOR MECHANICAL ENGINEERING,2022,46(01):47-55+60.

2. There is a fundamental inconsistency in this manuscript: the title and main claims refer to a TiAl coating, yet the powder material used is AlMgSc (an aluminum alloy), not a TiAl powder. The TiAl intermetallic phases (TiAl, TiAl_2, Ti_2Al, TiAl_3, Ti_3Al, Ti_5Al_11) form in-situ through reactions between Al from the AlMgSc powder and Ti diffusing from the TC4 substrate. The authors must explicitly distinguish between the bulk coating (AlMgSc) and the interfacial reaction zone (TiAl intermetallics), and revise the terminology throughout the manuscript to avoid misleading readers.

Response：Thanks for your suggestion. This paper fabricated a TiAl protective coating on the surface of titanium alloy using AlMgSc powder. The principle is that the Al in the powder undergoes a thermochromic reaction with the titanium alloy to form a TiAl intermetallic compound. The outermost AlMgSc alloy layer of the coating does not have the function of improving the surface properties of the base titanium alloy. We have revised the relevant terms throughout the manuscript. In Section 3.1, we have particularly emphasized: The interface reaction zone is the selected titanium-aluminum intermetallic compound protective coating. This is done to avoid misleading the readers and to mark the revised content in red.

3. The authors invoke the Gibbs free energy equation (ΔG = ΔH − TΔS) to explain the phase formation mechanism at each laser power level. However, not a single numerical value of ΔG, ΔH, or ΔS is presented to support these arguments. Statements such as "ΔG < 0 and sufficient nucleation driving force" without quantitative thermodynamic calculations render these arguments highly speculative. The authors are strongly advised to perform thermodynamic calculations using CALPHAD-based software (e.g., Thermo-Calc or FactSage) or to cite quantified literature data for each reported phase to substantiate their mechanistic claims.

Response：Thanks for your suggestion, the authors agree with the reviewers' opinion that conducting thermodynamic calculations using software based on CALPHAD (such as Thermo-Calc or FactSage) would be beneficial. We understand this. However, due to the limitations of laboratory conditions, this test experiment cannot be carried out at present. We will pay more attention to improving the experimental prototype and will do our best to incorporate thermodynamic calculations for confirmatory analysis in subsequent experi

Attachment Submitted filename: Response to Reviewers.docx

# Peer review: Decision Letter 1

**DOI:** 10.1371/journal.pone.0356182.r003

**Authors:** Hari Murthy

3 Jun 2026

Dear Dr. Yong,

Please submit your revised manuscript by Jul 18 2026 11:59PM. If you will need more time than this to complete your revisions, please reply to this message or contact the journal office at plosone@plos.org. When you're ready to submit your revision, log on to https://www.editorialmanager.com/pone/ and select the 'Submissions Needing Revision' folder to locate your manuscript file.

- A letter that responds to each point raised by the academic editor and reviewer(s). You should upload this letter as a separate file labeled 'Response to Reviewers'.
- A marked-up copy of your manuscript that highlights changes made to the original version. You should upload this as a separate file labeled 'Revised Manuscript with Track Changes'.
- An unmarked version of your revised paper without tracked changes. You should upload this as a separate file labeled 'Manuscript'.

If you would like to make changes to your financial disclosure, please include your updated statement in your cover letter. Guidelines for resubmitting your figure files are available below the reviewer comments at the end of this letter.

If applicable, we recommend that you deposit your laboratory protocols in protocols.io to enhance the reproducibility of your results. Protocols.io assigns your protocol its own identifier (DOI) so that it can be cited independently in the future. For instructions see: https://journals.plos.org/plosone/s/submission-guidelines#loc-laboratory-protocols. Additionally, PLOS ONE offers an option for publishing peer-reviewed Lab Protocol articles, which describe protocols hosted on protocols.io. Read more information on sharing protocols at https://plos.org/protocols?utm_medium=editorial-email&utm_source=authorletters&utm_campaign=protocols.

As the corresponding author, your ORCID iD is verified in the submission system and will appear in the published article. PLOS supports the use of ORCID, and we encourage all coauthors to register for an ORCID iD and use it as well. Please encourage your coauthors to verify their ORCID iD within the submission system before final acceptance, as unverified ORCID iDs will not appear in the published article. *Only*  the individual author can complete the verification step; PLOS staff *cannot*  verify ORCID iDs on behalf of authors.

We look forward to receiving your revised manuscript.

Kind regards,

Hari Murthy, Ph.D.

Academic Editor

PLOS One

Journal Requirements:

If the reviewer comments include a recommendation to cite specific previously published works, please review and evaluate these publications to determine whether they are relevant and should be cited. There is no requirement to cite these works unless the editor has indicated otherwise.

Additional Editor Comments:

Most of the reviewer comments has been addressed satisfactorily to a great extent- however there is still some ambiguity regarding the technical content and the paper in its current form is not yet publishable. Request you to kindly go through the reviewer comments and make necessary changes.

[Note: HTML markup is below. Please do not edit.]

Reviewers' comments:

Reviewer's Responses to Questions

**Comments to the Author**

Reviewer #1: All comments have been addressed

Reviewer #2: All comments have been addressed

Reviewer #4: (No Response)

**********

2. Is the manuscript technically sound, and do the data support the conclusions?

Reviewer #1: Yes

Reviewer #2: Yes

Reviewer #4: Yes

**********

3. Has the statistical analysis been performed appropriately and rigorously?

Reviewer #1: Yes

Reviewer #2: Yes

Reviewer #4: No

**********

4. Have the authors made all data underlying the findings in their manuscript fully available?

The PLOS Data policy

Reviewer #1: Yes

Reviewer #2: Yes

Reviewer #4: Yes

**********

5. Is the manuscript presented in an intelligible fashion and written in standard English?

Reviewer #1: Yes

Reviewer #2: Yes

Reviewer #4: No

**********

Reviewer #1: (No Response)

Reviewer #2: (No Response)

Reviewer #4: The manuscript entitled “Effect of Laser Power Variation on Microstructure and Properties of Selective Laser Melting Coatings Deposited on TC4 Titanium Alloy” investigates the influence of laser power on the formation quality, microstructure, phase composition, elemental distribution, and hardness of coatings fabricated on TC4 titanium alloy substrates using selective laser melting (SLM). The authors employed SEM, EDS, XRD, and microhardness measurements to compare coatings fabricated under laser powers ranging from 350 W to 380 W. The results indicate that insufficient laser power leads to incomplete melting and severe cracking, while excessive power causes overheating-induced cracking, whereas an intermediate power of 370 W produces improved metallurgical bonding and the highest hardness value. The topic is practically relevant for titanium alloy surface engineering and aerospace applications, and the revised manuscript shows improvement in formatting, terminology consistency, figure presentation, and experimental description. The authors have also addressed several reviewer comments related to abstract clarity, figure labeling, statistical presentation of hardness values, and manuscript organization. However, despite these improvements, several important scientific concerns remain insufficiently addressed. The overall novelty of the work remains limited because the observed dependence of coating quality on laser energy density largely confirms already established SLM behavior. In addition, the distinction between the AlMgSc feedstock and the TiAl intermetallic reaction zone is still not fully clarified throughout the manuscript. The mechanistic discussion continues to rely heavily on qualitative Gibbs free energy arguments without quantitative thermodynamic calculations or CALPHAD analysis, making several conclusions speculative. Furthermore, the study only evaluates hardness and microstructure, while no adhesion, wear, oxidation, or corrosion testing is provided to support the claimed protective performance of the coatings. The statistical rigor is also still relatively weak, with limited discussion of repeatability and uncertainty. Therefore, although the manuscript has improved after revision and may be suitable for publication after further revision, several major scientific issues should still be addressed before acceptance.

Recommendation: Major Revision

Major comments:

1. The novelty and scientific contribution of the work should be more clearly articulated. The observed trend of insufficient energy input causing poor bonding and excessive energy causing cracking is already well established in the SLM literature. The authors should explicitly explain what new insight this study contributes beyond parameter optimization.

2. The manuscript still contains ambiguity regarding the material system. The feedstock material is AlMgSc powder, while the manuscript repeatedly refers to the product as a “TiAl coating.” The authors should clearly distinguish the deposited AlMgSc layer, the Ti-Al interfacial reaction zone, and the intermetallic transition region throughout the manuscript.

3. The mechanistic discussion remains largely qualitative. The repeated use of Gibbs free energy arguments without quantitative thermodynamic calculations, phase diagram analysis, or CALPHAD support weakens the scientific rigor of the discussion. At minimum, the speculative nature of these interpretations should be explicitly acknowledged and moderated.

4. The manuscript claims protective coating functionality for aerospace applications, yet no wear resistance, oxidation resistance, corrosion resistance, or adhesion testing is presented. Hardness alone is insufficient to demonstrate coating performance. The engineering claims should therefore be moderated unless additional functional testing is included.

5. The statistical rigor should be improved further. The manuscript now includes standard deviation values for hardness measurements, which is appreciated; however, the number of independently fabricated samples per condition remains unclear. The authors should clarify the repeatability of the experiments and provide statistical treatment for coating thickness measurements as well.

6. The literature review should be revised to focus more specifically on TiAl intermetallic coatings on titanium substrates and related interfacial reaction systems. Many currently cited references are generic SLM studies with limited direct relevance to the present material system.

Minor comments:

1. The manuscript still contains several long and grammatically awkward sentences, particularly in Sections 3.1 and 3.2. Additional English editing is recommended.

2. Figure quality has improved, but several SEM images still require clearer contrast and labeling for publication quality.

3. The discussion of the non-monotonic coating thickness trend should be strengthened further with a more detailed explanation of melt pool dynamics and possible evaporation or recoil pressure effects.

4. The terminology describing the coating should be made fully consistent throughout the manuscript.

**********

what does this mean?). If published, this will include your full peer review and any attached files.

If you choose “no”, your identity will remain anonymous but your review may still be made public.

**Do you want your identity to be public for this peer review?**  For information about this choice, including consent withdrawal, please see our Privacy Policy

Reviewer #1: **Yes:** Ahmed H. Maamoun

Reviewer #2: No

Reviewer #4: No

**********

[NOTE: If reviewer comments were submitted as an attachment file, they will be attached to this email and accessible via the submission site. Please log into your account, locate the manuscript record, and check for the action link "View Attachments". If this link does not appear, there are no attachment files.]

To ensure your figures meet our technical requirements, please review our figure guidelines: https://journals.plos.org/plosone/s/figures

You may also use PLOS’s free figure tool, NAAS, to help you prepare publication quality figures: https://journals.plos.org/plosone/s/figures#loc-tools-for-figure-preparation.

NAAS will assess whether your figures meet our technical requirements by comparing each figure against our figure specifications.

# Author comment: Author response to Decision Letter 2

**DOI:** 10.1371/journal.pone.0356182.r004

10 Jun 2026

The authors would like to thank the editors and reviewers for the careful consideration that they have given to this work. The paper has been revised taking into consideration the reviewers’ comments. As a result, the material in the paper has been clarified and improved, and hopefully it is now acceptable for publication.

1. The novelty and scientific contribution of the work should be more clearly articulated. The observed trend of insufficient energy input causing poor bonding and excessive energy causing cracking is already well established in the SLM literature. The authors should explicitly explain what new insight this study contributes beyond parameter optimization.

Response：Thanks very much for your comments, which are very helpful for us to improve the manuscript.In the concluding part of the introduction of the manuscript, the authors revised the main significance of this study, highlighting the novelty and scientific contribution of the research, and explaining some new insights it has brought beyond parameter optimization. And it is marked in red.

2. The manuscript still contains ambiguity regarding the material system. The feedstock material is AlMgSc powder, while the manuscript repeatedly refers to the product as a “TiAl coating.” The authors should clearly distinguish the deposited AlMgSc layer, the Ti-Al interfacial reaction zone, and the intermetallic transition region throughout the manuscript.

Response：We sincerely thanks the reviewer for the valuable suggestions. The authors have made modifications to the TiAl coating in the manuscript, changing it to a multi-alloy composite coating or a composite coating. In Section 3.1, the analysis of the coating structure proposed that: the outer layer of the coating is the deposited AlMgSc layer, and the interface between the coating and the substrate is the Ti-Al reaction zone and the intermetallic compound transformation zone.

3. The mechanistic discussion remains largely qualitative. The repeated use of Gibbs free energy arguments without quantitative thermodynamic calculations, phase diagram analysis, or CALPHAD support weakens the scientific rigor of the discussion. At minimum, the speculative nature of these interpretations should be explicitly acknowledged and moderated.

Response：Thank you for pointing this out. We agree with this comment. I have revised the analysis of XRD in Section 3.2. I have reduced the discussion of the Gibbs free energy and acknowledged and moderately limited the speculative nature of these explanations. And explain the reasons for the formation of the new phase from the perspective of thermodynamic analysis. Marked in red.

4. The manuscript claims protective coating functionality for aerospace applications, yet no wear resistance, oxidation resistance, corrosion resistance, or adhesion testing is presented. Hardness alone is insufficient to demonstrate coating performance. The engineering claims should therefore be moderated unless additional functional testing is included.

Response：We sincerely appreciate the valuable comments from the reviewers, as these comments helped us improve the quality of the manuscript. We would like to express our gratitude to the reviewers for their valuable suggestions. Due to the disassembly of the samples and the return of the leased testing equipment, we are unable to provide the test results for wear resistance, oxidation resistance, corrosion resistance or adhesion. In accordance with the reviewers' suggestions, we have appropriately revised the engineering statement at the end of the introduction section of the manuscript. For example:“providing new analytical ideas and technical support for the improvement of titanium alloy surface properties and the design of coatings, but also provides reliable theoretical and experimental basis for the additive manufacturing preparation of protective coatings for aerospace titanium alloys.” At the same time, the expressions related to the engineering aspect in the conclusion part of the abstract were revised.

5. The statistical rigor should be improved further. The manuscript now includes standard deviation values for hardness measurements, which is appreciated; however, the number of independently fabricated samples per condition remains unclear. The authors should clarify the repeatability of the experiments and provide statistical treatment for coating thickness measurements as well.

Response：Thanks for your suggestion. In the final part of the second chapter of the manuscript, the authors demonstrate the reproducibility of the experiment. For instance, in this experiment, three parallel samples were independently prepared for each laser power condition, ensuring the reliability and repeatability of the experimental data. Meanwhile, in Section 3.1 of the manuscript, we have added a statistical analysis of the measurement results of the coating thickness. For example:For each laser power group, three independent parallel samples were prepared. Each sample was cut to prepare cross-sectional metallographic specimens. For each sample section, three observation areas were selected at equal intervals, and 5 coating thickness readings were randomly collected from each area. The average coating thickness value of each group was calculated as the final coating thickness value.

6. The literature review should be revised to focus more specifically on TiAl intermetallic coatings on titanium substrates and related interfacial reaction systems. Many currently cited references are generic SLM studies with limited direct relevance to the present material system.

Response:Thanks. The authors have removed some of the commonly cited references from the SLM research, such as those in references [24]-[33] and [35], [36]、[39]、[41]、[42], etc. And the labels of other references in the text have been revised. The modified parts are marked in red.

Minor comments:

1. The manuscript still contains several long and grammatically awkward sentences, particularly in Sections 3.1 and 3.2. Additional English editing is recommended.

Response：Thank you for pointing this out. The authors have refined the long sentences and those with improper grammar in the manuscript, especially in Sections 3.1 and 3.2, and marked them in red.

2. Figure quality has improved, but several SEM images still require clearer contrast and labeling for publication quality.

Response：Thanks for your suggestion. We have made modifications to the pictures in the manuscript. We have re-drawn the annotations for pictures 2 and 3, and improved the clarity of pictures 7 and 8. The pictures in the entire manuscript are much clearer.

3. The discussion of the non-monotonic coating thickness trend should be strengthened further with a more detailed explanation of melt pool dynamics and possible evaporation or recoil pressure effects.

Response：Thanks for your suggestion. In Section 3.2 of the manuscript, we have added an additional section in the coating SEM analysis, focusing on discussing the possible reasons for the non-monotonic coating thickness trend, providing a detailed explanation of the molten pool dynamics and the possible effects of evaporation or recoil pressure. Marked in red.

4. The terminology describing the coating should be made fully consistent throughout the manuscript.

Response：We sincerely thanks the reviewer for the valuable suggestions. We have standardized and unified the terms used to describe the coating throughout the entire manuscript. We have also revised the previous TiAl coating, for example: composite coating.

Attachment Submitted filename: Response_to_Reviewers_auresp_2.docx

# Peer review: Decision Letter 2

**DOI:** 10.1371/journal.pone.0356182.r005

**Authors:** Hari Murthy

7 Jul 2026

Dear Dr. Yong,

Thank you for submitting your manuscript to PLOS ONE. After careful consideration, we feel that it has merit but does not fully meet PLOS ONE’s publication criteria as it currently stands. Therefore, we invite you to submit a revised version of the manuscript that addresses the points raised during the review process.

Please submit your revised manuscript by Aug 21 2026 11:59PM. If you will need more time than this to complete your revisions, please reply to this message or contact the journal office at plosone@plos.org. When you're ready to submit your revision, log on to https://www.editorialmanager.com/pone/ and select the 'Submissions Needing Revision' folder to locate your manuscript file.

- A letter that responds to each point raised by the academic editor and reviewer(s). You should upload this letter as a separate file labeled 'Response to Reviewers'.
- A marked-up copy of your manuscript that highlights changes made to the original version. You should upload this as a separate file labeled 'Revised Manuscript with Track Changes'.
- An unmarked version of your revised paper without tracked changes. You should upload this as a separate file labeled 'Manuscript'.

If you would like to make changes to your financial disclosure, please include your updated statement in your cover letter. Guidelines for resubmitting your figure files are available below the reviewer comments at the end of this letter.

If applicable, we recommend that you deposit your laboratory protocols in protocols.io to enhance the reproducibility of your results. Protocols.io assigns your protocol its own identifier (DOI) so that it can be cited independently in the future. For instructions see: https://journals.plos.org/plosone/s/submission-guidelines#loc-laboratory-protocols. Additionally, PLOS ONE offers an option for publishing peer-reviewed Lab Protocol articles, which describe protocols hosted on protocols.io. Read more information on sharing protocols at https://plos.org/protocols?utm_medium=editorial-email&utm_source=authorletters&utm_campaign=protocols.

As the corresponding author, your ORCID iD is verified in the submission system and will appear in the published article. PLOS supports the use of ORCID, and we encourage all coauthors to register for an ORCID iD and use it as well. Please encourage your coauthors to verify their ORCID iD within the submission system before final acceptance, as unverified ORCID iDs will not appear in the published article. *Only*  the individual author can complete the verification step; PLOS staff *cannot*  verify ORCID iDs on behalf of authors.

We look forward to receiving your revised manuscript.

Kind regards,

Hari Murthy, Ph.D.

Academic Editor

PLOS One

**Journal Requirements:**

1. If the reviewer comments include a recommendation to cite specific previously published works, please review and evaluate these publications to determine whether they are relevant and should be cited. There is no requirement to cite these works unless the editor has indicated otherwise.

2. Please review your reference list to ensure that it is complete and correct. If you have cited papers that have been retracted, please include the rationale for doing so in the manuscript text, or remove these references and replace them with relevant current references. Any changes to the reference list should be mentioned in the rebuttal letter that accompanies your revised manuscript. If you need to cite a retracted article, indicate the article’s retracted status in the References list and also include a citation and full reference for the retraction notice.

[Note: HTML markup is below. Please do not edit.]

Reviewers' comments:

Reviewer's Responses to Questions

**Comments to the Author**

Reviewer #1: All comments have been addressed

Reviewer #2: All comments have been addressed

Reviewer #4: All comments have been addressed

**********

2. Is the manuscript technically sound, and do the data support the conclusions?

Reviewer #1: Yes

Reviewer #2: Yes

Reviewer #4: Yes

**********

3. Has the statistical analysis been performed appropriately and rigorously?

Reviewer #1: Yes

Reviewer #2: Yes

Reviewer #4: Yes

**********

4. Have the authors made all data underlying the findings in their manuscript fully available?

The PLOS Data policy

Reviewer #1: Yes

Reviewer #2: Yes

Reviewer #4: Yes

**********

5. Is the manuscript presented in an intelligible fashion and written in standard English?

Reviewer #1: Yes

Reviewer #2: Yes

Reviewer #4: Yes

**********

**Reviewer #1:** The revised manuscript is significantly improved. The review comments and recommendations have been addressed. However, some issues still need to be considered as follows:

- In Section 2, the powder material name should be clearly stated before presenting the powder characteristics such as particle size distribution. Also, it is very crucial to illustrate the powder morphology and particle shape.

- In subsection 3.2 related to phase analysis; the manuscript text should illustrate more analysis and discussion for the data presented in Table 3 related to the significant variation of elements' wt% of the EDS results throughout the manuscript text, and the discussion should be supported by relevant references.

- The directions related to the building direction should be labelled on all Figures containing SEM and microscopic images.

- The references of the presented data in Figure 4 should be cited and illustrated in a separate column in the Table.

**Reviewer #2:**  (No Response)

**Reviewer #4:** The revised manuscript presents a systematic experimental investigation of the effect of laser power on the microstructure, phase evolution, elemental distribution, and microhardness of selective laser melting (SLM) coatings deposited on TC4 titanium alloy. The experimental design is straightforward, and the combination of SEM, EDS, XRD, and hardness measurements provides a comprehensive characterization of the coatings. The manuscript is generally well organized, and the experimental results consistently demonstrate that a laser power of 370 W produces the most favorable coating quality with improved metallurgical bonding and the highest microhardness.

The current version has been substantially improved in terms of experimental description, statistical information, and presentation of the results. The distinction between the deposited layer and the interfacial reaction zone is much clearer, the experimental repeatability is better documented, and the discussion has been strengthened with additional analysis of coating thickness evolution and microstructural development. Overall, the study is technically sound and provides useful experimental data for understanding the influence of laser power on this specific coating system.

I only have a few minor comments that should be addressed before publication.

The English writing has improved, but the manuscript would still benefit from careful language editing. Several sentences remain grammatically awkward or unnecessarily long, particularly in the Introduction and Discussion sections. A final proofreading by a fluent English speaker is recommended to improve readability.

The manuscript occasionally overstates the significance of its findings. Expressions such as "fills the existing research gap," "forms a new understanding," and "provides theoretical support" should be moderated. The work represents a systematic experimental study of laser power optimization for a particular material system, and the conclusions should reflect this scope more appropriately.

Some mechanistic explanations remain largely qualitative. Discussions involving Gibbs free energy, recoil pressure, keyhole transition, and phase evolution are reasonable interpretations but are not supported by quantitative thermodynamic calculations or numerical simulations. The corresponding statements should therefore be presented as proposed mechanisms rather than definitive conclusions.

Although the terminology has been improved, some inconsistency remains in the description of the coating. Terms such as "TiAl coating," "composite coating," and "protective coating" are still used interchangeably in several sections. Using consistent terminology throughout the manuscript would improve clarity.

Finally, several minor typographical and formatting issues remain, including duplicated words in the Abstract, occasional spacing inconsistencies, and minor formatting problems for units and symbols. These should be corrected during the final proofreading stage.

Overall, the manuscript is technically sound, the experimental methodology is appropriate, and the conclusions are generally supported by the presented results. The remaining issues are minor editorial revisions that do not require additional experiments. Therefore, I recommend **acceptance after minor revision**.

**********

what does this mean?). If published, this will include your full peer review and any attached files.

If you choose “no”, your identity will remain anonymous but your review may still be made public.

**Do you want your identity to be public for this peer review?**  For information about this choice, including consent withdrawal, please see our Privacy Policy

Reviewer #1: **Yes:** Ahmed H. Maamoun

Reviewer #2: No

Reviewer #4: No

**********

[NOTE: If reviewer comments were submitted as an attachment file, they will be attached to this email and accessible via the submission site. Please log into your account, locate the manuscript record, and check for the action link "View Attachments". If this link does not appear, there are no attachment files.]

To ensure your figures meet our technical requirements, please review our figure guidelines: https://journals.plos.org/plosone/s/figures

You may also use PLOS’s free figure tool, NAAS, to help you prepare publication quality figures: https://journals.plos.org/plosone/s/figures#loc-tools-for-figure-preparation.

NAAS will assess whether your figures meet our technical requirements by comparing each figure against our figure specifications.

# Author comment: Author response to Decision Letter 3

**DOI:** 10.1371/journal.pone.0356182.r006

20 Jul 2026

Journal Requirements:

1.If the reviewer comments include a recommendation to cite specific previously published works, please review and evaluate these publications to determine whether they are relevant and should be cited. There is no requirement to cite these works unless the editor has indicated otherwise.

Response：Thanks very much for your comments, which are very helpful for us to improve the manuscript. The reviewers' comments did not mention the suggestion to cite specific published literature, and the authors themselves will not cite any specific published works.

2. Please review your reference list to ensure that it is complete and correct. If you have cited papers that have been retracted, please include the rationale for doing so in the manuscript text, or remove these references and replace them with relevant current references. Any changes to the reference list should be mentioned in the rebuttal letter that accompanies your revised manuscript. If you need to cite a retracted article, indicate the article’s retracted status in the References list and also include a citation and full reference for the retraction notice.

Response：Thank you for pointing this out. We agree with this comment. After reviewing the references, we found that reference [9] has been retracted, so it has been removed and replaced with the most recent relevant reference.

Reviewer #1: The revised manuscript is significantly improved. The review comments and recommendations have been addressed. However, some issues still need to be considered as follows:

1. In Section 2, the powder material name should be clearly stated before presenting the powder characteristics such as particle size distribution. Also, it is very crucial to illustrate the powder morphology and particle shape.

Response：Thanks very much for your comments, which are very helpful for us to improve the manuscript. In the initial part of Section 2 of the manuscript, the authors added Figure 1. Figure 1 (a) shows the surface morphology of AlMgSc particles observed by the Thermo Scientific Quattro scanning electron microscope (SEM), clearly presenting the morphology and shape of the powder, and the AlMgSc matrix powder maintains a high degree of sphericity. Figure 1 (b) presents the powder particle size distribution results measured by the laser particle size analyzer, introducing the powder characteristics (such as particle size distribution).

2.In subsection 3.2 related to phase analysis; the manuscript text should illustrate more analysis and discussion for the data presented in Table 3 related to the significant variation of elements' wt% of the EDS results throughout the manuscript text, and the discussion should be supported by relevant references.

Response：Thank you for pointing this out. We agree with this comment. In Section 3.2, the authors further analyzed and discussed the significant changes in the data presented in Figure 7 (now renamed as Figure 9) and Table 3. They analyzed the changes in the mass percentages of each element in the electron energy spectrum (EDS) results, and also added appropriate references. The modified content is marked in red.

3. The directions related to the building direction should be labelled on all Figures containing SEM and microscopic images.

Response：We sincerely thanks the reviewer for the valuable suggestions. The authors have added the building directions to all the figures in the manuscript that contain scanning electron microscopy and microscopic images. In Section 3.1, the meaning of the abbreviation "BD" is specifically explained, for example, "building direction (BD) refers to the construction direction."

4.The references of the presented data in Figure 4 should be cited and illustrated in a separate column in the Table.

Response：Thanks for your suggestion, the data in Picture 4 are the data used in this experiment. The purpose is to show the influence of different laser powers on the coating quality in this experiment. No data from other references were used or cited in the picture.

Reviewer #4: The revised manuscript presents a systematic experimental investigation of the effect of laser power on the microstructure, phase evolution, elemental distribution, and microhardness of selective laser melting (SLM) coatings deposited on TC4 titanium alloy. The experimental design is straightforward, and the combination of SEM, EDS, XRD, and hardness measurements provides a comprehensive characterization of the coatings. The manuscript is generally well organized, and the experimental results consistently demonstrate that a laser power of 370 W produces the most favorable coating quality with improved metallurgical bonding and the highest microhardness.

The current version has been substantially improved in terms of experimental description, statistical information, and presentation of the results. The distinction between the deposited layer and the interfacial reaction zone is much clearer, the experimental repeatability is better documented, and the discussion has been strengthened with additional analysis of coating thickness evolution and microstructural development. Overall, the study is technically sound and provides useful experimental data for understanding the influence of laser power on this specific coating system.

I only have a few minor comments that should be addressed before publication.

1.The English writing has improved, but the manuscript would still benefit from careful language editing. Several sentences remain grammatically awkward or unnecessarily long, particularly in the Introduction and Discussion sections. A final proofreading by a fluent English speaker is recommended to improve readability.

Response： We sincerely appreciate the valuable comments from the reviewers, as these comments helped us improve the quality of the manuscript. The authors have thoroughly reviewed the manuscript in response to the reviewers' comments, making every effort to improve the content and completing numerous targeted revisions. All the modifications made this time are aimed at addressing issues such as grammatical incoherence or unnecessary length in some sentences, with a focus on the introduction and discussion sections. The adjusted content is marked in red font throughout the revised manuscript. We will not list each item separately here. We sincerely thank the editor and all the reviewers for spending their valuable time carefully reviewing this article. We also greatly appreciate the professional guidance provided by all of you. We earnestly request that the editor and reviewers approve these revisions.

2.The manuscript occasionally overstates the significance of its findings. Expressions such as "fills the existing research gap," "forms a new understanding," and "provides theoretical support" should be moderated. The work represents a systematic experimental study of laser power optimization for a particular material system, and the conclusions should reflect this scope more appropriately.

Response：We sincerely appreciate your objective, fair, and meticulous review comments. The authors removed "fills the existing research gap" at the end of the abstract and replaced it with "This research provides certain technical references and theoretical support for the preparation of TiAl coatings on the surface of aerospace titanium alloys."

The authors removed the sentence "This study breaks away from the traditional framework of single-component coating research" in the conclusion section, and changed the following: "Provide new theoretical support and practical references for the parameter optimization and performance regulation of titanium alloy surface strengthening processes." to: "It provides certain theoretical support and reference for the parameter optimization and performance regulation of the TiAl coating on the titanium alloy surface."

3.Some mechanistic explanations remain largely qualitative. Discussions involving Gibbs free energy, recoil pressure, keyhole transition, and phase evolution are reasonable interpretations but are not supported by quantitative thermodynamic calculations or numerical simulations. The corresponding statements should therefore be presented as proposed mechanisms rather than definitive conclusions.

Response：Thank you for your reminder. In Section 3.1 of the manuscript, the authors added some explanations, stating that the corresponding statements represent the proposed mechanism, such as: "The possible reasons might be,...". Some of the proposed mechanisms in the manuscript were made based on explanations derived from other referenced literature. In Section 3.2, replace: "The thermodynamic calculation showed that at this time," with "According to thermodynamic calculations,". And mark the modified parts in red.

4.Although the terminology has been improved, some inconsistency remains in the description of the coating. Terms such as "TiAl coating," "composite coating," and "protective coating" are still used interchangeably in several sections. Using consistent terminology throughout the manuscript would improve clarity.

Response：Thank you for your reminder. The authors have uniformly referred to "TiAl coating," "composite coating," and "protective coating" as "TiAl coating."

5.Finally, several minor typographical and formatting issues remain, including duplicated words in the Abstract, occasional spacing inconsistencies, and minor formatting problems for units and symbols. These should be corrected during the final proofreading stage.

Response：Thanks for your suggestion, the authors have carefully checked the minor layout and formatting issues in the manuscript, corrected the minor formatting problems of units and symbols, and marked them in red.

Overall, the manuscript is technically sound, the experimental methodology is appropriate, and the conclusions are generally supported by the presented results. The remaining issues are minor editorial revisions that do not require additional experiments. Therefore, I recommend **acceptance after minor revision**.

We again extend our deepest appreciation to the editorial team and reviewers for your careful and professional guidance. All revisions in this manuscript have been completed rigorously following your detailed suggestions. Please do not hesitate to share any further revisions or adjustments if the paper still requires polishing. We eagerly await your feedback.

Attachment Submitted filename: Response_to_Reviewers_auresp_3.docx

# Peer review: Decision Letter 3

**DOI:** 10.1371/journal.pone.0356182.r007

**Authors:** Hari Murthy

30 Jul 2026

Effect of Laser Power Variation on Microstructure and Properties of Selective Laser Melting Coatings Deposited on TC4 Titanium Alloy

PONE-D-26-09790R3

Dear Dr. Yong,

We’re pleased to inform you that your manuscript has been judged scientifically suitable for publication and will be formally accepted for publication once it meets all outstanding technical requirements.

Within one week, you’ll receive an e-mail detailing the required amendments. When these have been addressed, you’ll receive a formal acceptance letter and your manuscript will be scheduled for publication.

An invoice will be generated when your article is formally accepted. Please note, if your institution has a publishing partnership with PLOS and your article meets the relevant criteria, all or part of your publication costs will be covered. Please make sure your user information is up-to-date by logging into Editorial Manager at Editorial Manager® and clicking the ‘Update My Information' link at the top of the page. For questions related to billing, please contact billing support.

If your institution or institutions have a press office, please notify them about your upcoming paper to help maximize its impact. If they’ll be preparing press materials, please inform our press team as soon as possible -- no later than 48 hours after receiving the formal acceptance. Your manuscript will remain under strict press embargo until 2 pm Eastern Time on the date of publication. For more information, please contact onepress@plos.org.

Kind regards,

Hari Murthy, Ph.D.

Academic Editor

PLOS One

Additional Editor Comments (optional):

Reviewers' comments:

Reviewer's Responses to Questions

**Comments to the Author**

Reviewer #1: All comments have been addressed

Reviewer #4: All comments have been addressed

**********

2. Is the manuscript technically sound, and do the data support the conclusions?

Reviewer #1: Yes

Reviewer #4: Yes

**********

3. Has the statistical analysis been performed appropriately and rigorously?

Reviewer #1: Yes

Reviewer #4: Yes

**********

4. Have the authors made all data underlying the findings in their manuscript fully available?

The PLOS Data policy

Reviewer #1: Yes

Reviewer #4: Yes

**********

5. Is the manuscript presented in an intelligible fashion and written in standard English?

Reviewer #1: Yes

Reviewer #4: Yes

**********

Reviewer #1: The review comments and recommendations have been addressed. As a minor comment, the future perspective should be added to the conclusion.

Reviewer #4: All issues have been addressed properly by the authors, I would like to recommend accept this manuscript in current form.

**********

what does this mean?). If published, this will include your full peer review and any attached files.

If you choose “no”, your identity will remain anonymous but your review may still be made public.

**Do you want your identity to be public for this peer review?**  For information about this choice, including consent withdrawal, please see our Privacy Policy

Reviewer #1: **Yes:** Ahmed H. Maamoun

Reviewer #4: No

**********

# Editor report: Acceptance letter

**DOI:** 10.1371/journal.pone.0356182.r008

**Authors:** Hari Murthy

PONE-D-26-09790R3

PLOS One

Dear Dr. Li,

I'm pleased to inform you that your manuscript has been deemed suitable for publication in PLOS One. Congratulations! Your manuscript is now being handed over to our production team.

At this stage, our production department will prepare your paper for publication. This includes ensuring the following:

* All references, tables, and figures are properly cited

* All relevant supporting information is included in the manuscript submission,

* There are no issues that prevent the paper from being properly typeset

You will receive further instructions from the production team, including instructions on how to review your proof when it is ready. Please keep in mind that we are working through a large volume of accepted articles, so please give us a few days to review your paper and let you know the next and final steps.

Lastly, if your institution or institutions have a press office, please let them know about your upcoming paper now to help maximize its impact. If they'll be preparing press materials, please inform our press team within the next 48 hours. Your manuscript will remain under strict press embargo until 2 pm Eastern Time on the date of publication. For more information, please contact onepress@plos.org.

You will receive an invoice from PLOS for your publication fee after your manuscript has reached the completed accept phase. If you receive an email requesting payment before acceptance or for any other service, this may be a phishing scheme. Learn how to identify phishing emails and protect your accounts at https://explore.plos.org/phishing.

If we can help with anything else, please email us at customercare@plos.org.

Thank you for submitting your work to PLOS One and supporting open access.

Kind regards,

PLOS One Editorial Office Staff

on behalf of

Dr. Hari Murthy

Academic Editor

PLOS One
